# Publication‐driven consistency in food web structures: Implications for comparative ecology

**DOI:** 10.1002/ecy.4467

**Published:** 2024-11-21

**Authors:** Chris Brimacombe, Korryn Bodner, Dominique Gravel, Shawn J. Leroux, Timothée Poisot, Marie‐Josée Fortin

**Affiliations:** ^1^ Ecology and Evolutionary Biology University of Toronto Toronto Ontario Canada; ^2^ MAP Centre for Urban Health Solutions, St. Michael's Hospital Unity Health Toronto Toronto Ontario Canada; ^3^ Département de Biologie Université de Sherbrooke Sherbrooke Québec Canada; ^4^ Department of Biology Memorial University of Newfoundland and Labrador St. John's Newfoundland and Labrador Canada; ^5^ Département de Sciences Biologiques Université de Montréal Montréal Québec Canada; ^6^ Quebec Centre for Biodiversity Science Montréal Québec Canada

**Keywords:** communities, methodology, networks, sampling, species interactions, subgraphs, trophic relationships

## Abstract

Large collections of freely available food webs are commonly reused by researchers to infer how biological or environmental factors influence the structure of ecological communities. Although reusing food webs expands sample sizes for community analysis, this practice also has significant drawbacks. As food webs are meticulously crafted by researchers for their own specific research endeavors and resulting publications (i.e., books and scientific articles), the structure of these webs inherently reflects the unique methodologies and protocols of their source publications. Consequently, combining food webs sourced from different publications without accounting for discrepancies that influence network structure may be problematic. Here, we investigate the determinants of structure in freely available food webs sourced from different publications, examining potential disparities that could hinder their effective comparison. Specifically, we quantify structural similarity across 274 commonly reused webs sourced from 105 publications using a subgraph technique. Surprisingly, we found no increased structural similarity between webs from the same ecosystem nor webs built using similar network construction methodologies. Yet, webs sourced from the same publication were very structurally similar with this degree of similarity increasing over time. As webs sourced from the same publication are typically sampled, constructed, and/or exposed to similar biological and environmental factors, publications likely holistically drive their own webs' structure to be similar. Our findings demonstrate the large effect that publications have on the structure of their own webs, which stymies inference when comparing the structure of webs sourced from different publications. We conclude by proposing different approaches that may be useful for reducing these publication‐related structural issues.

## INTRODUCTION

Food webs have a rich history in ecology (Dunne, 2006; Guimarães, 2020; May, 1983; Paine, 1988; Pringle & Hutchinson, 2020; Vázquez et al., 2022; Winemiller, 1990), as gaining insights into aspects of who eats whom has major implications for individual fitness, population dynamics, community structure, and evolutionary trajectories (e.g., Cohen et al., 1993; Pringle, 2020). Beyond depicting single interspecific interactions, whole food webs distill the complexity of intertwining feeding interactions into mathematically tractable networks (Guimarães, 2020), most often defining an ecological community's species as nodes and corresponding feeding interactions as links (Cartozo et al., 2005). In this regard, many community‐level properties have been inferred from network topology (Delmas et al., 2019; Fortin et al., 2021), including stability, resilience, and sustainability (Carpentier et al., 2021; Landi et al., 2018; McCann, 2011).

Much of our knowledge about food webs has relied on and continues to rely on reusing and comparing the structure of freely available empirical webs (Dunne, 2006; Goldwasser & Roughgarden, 1993; Poisot et al., 2021; Winemiller, 1990; Xing & Fayle, 2021). These freely available webs are networks previously constructed by researchers for their own publications (i.e., books and scientific articles) that have been uploaded onto online open‐access repositories. For example, the commonly used repository for obtaining species interaction networks, Web of Life (www.web-of-life.es), contains 316 networks originally sourced from purported 127 scientific articles, and other published and unpublished works. Because constructing webs de novo from original field data is extremely difficult, time‐consuming, and expensive (Borrelli et al., 2023; McLeod et al., 2021; Polis, 1991)—not to mention ancillary to the expertise of many researchers who work on food webs—freely available webs represent a cornucopia of community‐level data that can be used to investigate and test novel hypotheses (Kita et al., 2022; Xing & Fayle, 2021). Thus, webs from online repositories are routinely reused in meta‐analysis‐like studies under the implicit assumption that they are reliable and comparable, with little to no scrutiny of the properties of individual webs (Brimacombe et al., 2023; Pringle & Hutchinson, 2020).

Similar to other types of species interaction networks, the drivers influencing food web structure can be generally categorized into three broad classes: *biological and environmental factors*, *sampling strategies*, and *network construction methodologies* (Brimacombe et al., 2023). Of the three classes, ecologists are often most interested in *biological and environmental factors*, which include the biotic and abiotic drivers that shape interspecific interactions in communities (e.g., how the presence of transient seasonal predators alter food web structure; Brimacombe et al., 2021; how warmer temperatures reduce community stability; Zhao et al., 2023). The remaining two classes, *sampling strategies* and *network construction methodologies*, include drivers that researchers themselves create when attempting to measure and model the structure of food webs. First, the *sampling strategies* class consists of the study design decisions that shape web structure (e.g., amount of time; Kitching, 1987; Tavares‐Cromar & Williams, 1996, and area sampled; Galiana et al., 2022). These design decisions determine the particular suite of abiotic and biotic drivers acting on a community during a study period and influence the likelihood of detecting a given species or interaction. Second, the *network construction methodologies* class consists of the methodological approaches that influence food web structure via the decisions researchers make when assembling a web (e.g., node taxon resolution; Bodner et al., 2022; Gauzens et al., 2013; Hemprich‐Bennett et al., 2021, the focal organisms under study; Goldwasser & Roughgarden, 1993, and trophic interaction evidence [e.g., fecal, stomach, and direct observation; Hutchinson et al., 2022]).

**FIGURE 1:**
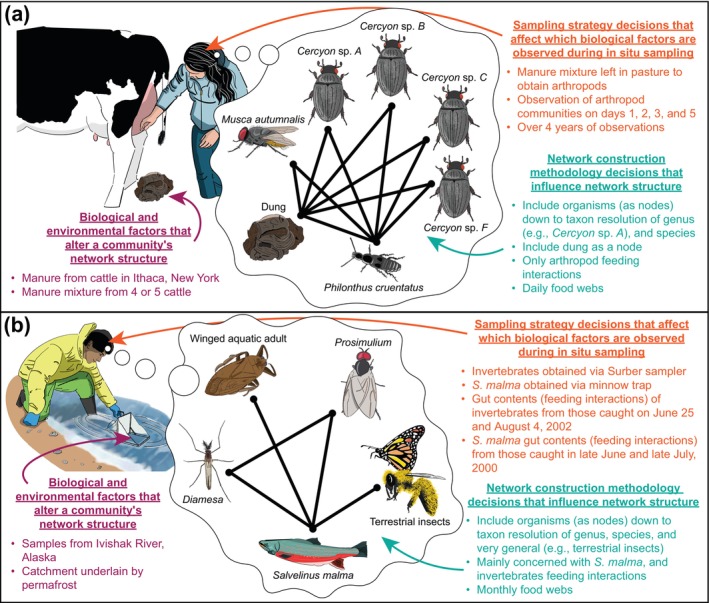
Example of how differences in the three classes of structure (i.e., *biological and environmental factors* [purple], *sampling strategies* [orange], and *network construction methodologies* [aqua]) cause food webs sourced from (a) Valiela (1974), and (b) Parker and Huryn (2006) to be very structurally different. Illustration reflects only a subset of nodes from each web (WEB200_ and WEB274_, respectively, from our food webs dataset, see Appendix S1: Table S7). Illustration by Chris Brimacombe.

When the *sampling strategies* and *network construction methodologies* do not effectively capture the species and trophic interactions of interest, the structure of an ecological community will be misrepresented (Carpentier et al., 2021; Goldwasser & Roughgarden, 1993; Hodkinson & Coulson, 2004; Paine, 1988; Pringle & Hutchinson, 2020). Even when the sampling and construction methodology accurately and precisely capture the species and trophic interactions in a web, inconsistencies in *biological and environmental factors*, *sampling strategies*, and *network construction methods* across webs can cause issues when comparing their structure (Brimacombe et al., 2023) (Figure 1). While it is possible to mitigate some of these structural discrepancies via network null models (Dormann et al., 2017; Farine, 2017), effective control relies on null models that are carefully posed for each analyzed network (Artzy‐Randrup et al., 2004). However, the creation of appropriate null models is especially challenging when networks sourced from different books and scientific articles (hereafter, *publication(s)*) are built using many different *sampling strategies* and *network construction methodologies*. For example, trophic interactions were measured by Valiela (1974) using direct observations of feeding habits and through feeding trials in petri dish arenas for their dung food webs, whereas Parker and Huryn (2006) used the gut contents of invertebrates and vertebrates manually caught in a river for evidence of interactions in their aquatic webs (Figure 1). Additionally, the careful tailoring of a null model or other controls may depend on additional information about each web, which is very rarely reported (Kita et al., 2022; Poisot, Baiser, et al., 2016), for example, the amount of area and time used to delineate the respective community. In some contexts, this information is perhaps even empirically unknowable given that many animals travel (with their gut contents) across large areas, effectively “importing” interactions into what might otherwise be small focal areas.

**FIGURE 2:**
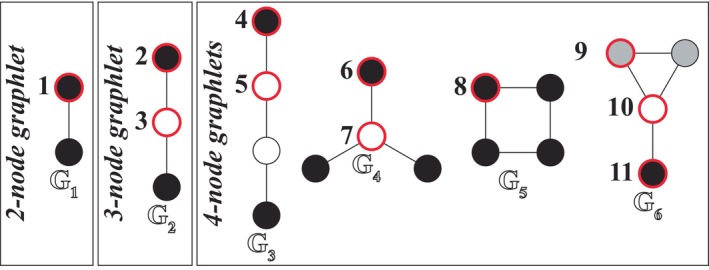
The six graphlets ($G_{i}$) consisting of two‐to‐four nodes and their respective automorphism orbits (“orbits,” nodes that are numerically labeled and outlined in red). Each unique shade in a graphlet corresponds to an orbit, which are nodes in the subgraph that are topologically identical.

One way to assess the combined contributions of *biological and environmental factors*, *sampling strategies*, and *network construction methodologies* on freely available food webs is by comparing the structure of webs originally sourced from the same publication to webs originally sourced from different publications. Often, multiple food webs are built for a single publication to evaluate the structure of (1) the same community across time (e.g., Tavares‐Cromar & Williams, 1996; Valiela, 1974), or (2) different communities across space (e.g., Thompson & Townsend, 2003, 2004). As multiple webs from the same publication likely experience similar *biological and environmental factors*, *sampling strategies*, and/or *network construction methodologies*, the structure of food webs from the same publication is likely constrained to be similar (Closs & Lake, 1994), which could cause issues when comparing webs from different publications. Potentially then, the frequency of subgraphs (i.e., smaller webs defined by their configuration of nodes and links, see Figure 2) could be overexpressed in food webs sourced from the same publication as previously shown for bipartite networks (Brimacombe et al., 2023). But unlike bipartite networks, links in food webs can connect any nodes in a given network, producing a greater number of possible subgraphs. This characteristic of food web structure could lead to different structural relationships when assessing the potential effect of publication.

In a more focused perspective to the possible role of publication, if the *network construction methodologies* class is a dominant driver of structure, as has been suggested for instance by Lin et al. (2022), then webs built using similar methodology should be much more structurally similar than a set of webs constructed using an assortment of construction methodologies. If so, then it may be best to only compare the structure of freely available food webs that were constructed using similar methodologies, for example, similar rules for assigning trophic links between nodes in webs. One approach to test this is to compare the structural similarity of aquatic food webs constructed via the commonly employed software tool Ecopath (Christensen & Walters, 2004) with those constructed not with Ecopath, but with various different methods (e.g., via gut contents, historical reports from the literature). Webs from Ecopath are developed using a mass‐balance model to describe the energy flow between compartments (e.g., species, functional groups), and require additional empirical information including biomass of prey and predators from the desired community (Baeta et al., 2011).

In this paper, we assess a collection of freely available food webs to identify and offer controls for potential structural issues that might otherwise limit their comparability in studies seeking to deduce ecological properties related to their structure. To this end, we compared the structural similarity in defined groups of freely available food webs to determine whether these sets of networks exhibit measurable structural disparities. We compared the similarity within and between groups of webs defined by their (1) ecosystem, (2) publication source, and (3) *network construction methodology*. With regard to (1), if food webs sampled from the same ecosystem (i.e., aquatic, aquatic and terrestrial, and terrestrial) are more structurally similar within their own ecosystem than across ecosystems, then structural differences exist that are driven by their experienced *biological and environmental factors*. For (2), if webs originally sourced from the same publication have much higher structural similarity than webs originally sourced from different publications, then there likely exists structural disparities driven by the networks' source publications that uniquely represent their network(s)' experienced *biological and environmental factors*, *sampling strategies*, and *network construction methodologies*. For (3), if aquatic food webs constructed via Ecopath are more structurally similar than aquatic webs constructed via an assortment of other methodologies, then structural disparities are likely due to differences in *network construction methodologies*.

## METHODS

### Food webs

All freely available food webs—originally sourced from different publications—that we reused for our own study came from four commonly cited network repositories (e.g., Barbosa & Siqueira, 2023; Carpentier et al., 2021): Global Web Database (www.canberra.edu.au/globalwebdb), Web of Life (www.web-of-life.es), Interaction Web Database (www.ecologia.ib.usp.br/iwdb), and GlobAL daTabasE of traits and food Web Architecture (https://idata.idiv.de/ddm/Data/ShowData/283). While each web had a putatively associated publication from which it was originally sourced that was provided by each repository, we discarded webs from our analyses when (1) further investigation indicated that a given web may not actually have been from the associated publication, or (2) we could not gain access to the original publication to confirm it as the source. We then manually inspected each food web's nodes to correct typographical errors. We list all changes we made to the food webs in Appendix S1: Section S1.1.

In addition, we imposed structural requirements for food webs to be included in our analyses. First, we included only multitrophic networks excluding bipartite networks. We excluded bipartite networks as they are likely to be structurally differently due to the requirement that links can only exist between nodes in the two different sets. Second, we chose to analyze food webs as undirected networks to eliminate the potential for incorrectly labeled directed interactions from influencing our results. Third, to reduce potential bias arising from using small networks (Michalska‐Smith & Allesina, 2019), we included only webs with at least 10 total nodes, comprising at least five unique consumer and resource nodes, respectively. Fourth, when a network was not fully connected, we only analyzed the giant component (i.e., the largest connected component; Fortin et al., 2021) of each food web given the uncertainty with regard to how to analyze disconnected networks (Brimacombe et al., 2023; Brimacombe, Bodner, Michalska‐Smith, et al., 2022). Under our criteria, of the 531 unfiltered food webs originally downloaded from the four repositories, we were left with 274 webs. From these, 191 were originally sourced from 22 publications that each provided multiple networks and 83 were sourced from one of the 83 publications that each provided only a single network. See Appendix S1: Table S7 for a list of all 274 food webs and their publication sources.

For each of the remaining 274 food webs obtained from the four aforementioned repositories, we identified the type of ecosystem from which it was sampled. Specifically, we classified webs into one of three ecosystem types (1) “aquatic,” which included marine, lakes, rivers, streams, and springs, (2) “aquatic and terrestrial,” which included salt marshes, ponds, bogs, mudflats, pitcher plants, and tree holes filled with water, or (3) “terrestrial,” which included sand dunes, forests, meadows, prairie, and farmlands. In total, 167 webs were classified as “aquatic,” 28 webs were classified as “aquatic and terrestrial,” and 79 webs were classified as “terrestrial.” Webs classified as “aquatic” were further investigated to determine whether Ecopath was the method used in their construction.

### Pairwise graphlet correlation distance‐11

We evaluated food web structural similarity using pairwise graphlet correlation distance‐11 (GCD‐11) (Yaveroğlu et al., 2014). We evaluated the structural similarity of networks using GCD‐11 because of its previous success in both correctly identifying groups of networks based on structure alone (Tantardini et al., 2019) and quantifying their structural differences (Brimacombe et al., 2023). Briefly, this heuristic method characterizes a web's structure by the correlations between the number of times each node in the web occupies each of the 11 orbit positions in 6 graphlets (Figure 2) and leverages this information to determine graphlet structural similarity between webs via orbit correlation patterns. See Appendix S1: Section S1.2 for a thorough and graphical example of this method.

While motifs (e.g., Milo et al., 2002; Stouffer et al., 2007) are another common subgraph technique used to analyze a single web's structure in ecology, GCD‐11 is able to additionally use graphlets to measure structural similarity across a set of webs without a network null model, and does so with the highest success compared to other approaches (Tantardini et al., 2019). Current network null models are challenging to use as benchmarks for empirical networks because the relationship between statistical significance and biological importance is unclear, and minor modifications to network null models can lead to large changes in significance (Artzy‐Randrup et al., 2004).

Generally, there are two steps involved when evaluating structural similarity between a set of webs using GCD‐11. In step one, the structure of each web is characterized using graphlets, which involves tallying the number of times each node in the web occupies each of the 11 orbit positions. For a node, this is represented by a vector with 11 entries (called a graphlet degree vector‐11) where each entry is the number of times the node occupies the respective orbit position. For a whole web consisting of *n* nodes, this is represented by a *n* × 11 matrix containing orbit counts on each *n* node. Next, Spearman's correlation between all possible combinations of orbit counts in the web is evaluated, that is, correlations between each 11 column vectors in the *n* × 11 matrix. The resulting output is a symmetric 11 × 11 matrix, referred to as a graphlet correlation matrix‐11 (GCM‐11), where entry (*i*, *j*) is the respective correlation between orbit vector counts of *i* and *j*. Simply put, these correlations are indicative of how nodes in the web act as interaction partners across graphlets. In step two, the pairwise Euclidean distance (Equation 1) between each web's GCM‐11 is evaluated:

(1) $$\text{pairwise}\mathrm{GCD}‐11\left(K_{i},K_{j}\right)=\sqrt{\sum_{n=1}^{11}\sum_{m=n+1}^{11}{\left(\mathrm{GCM}‐11_{K_{i}}\left(n,m\right)-\mathrm{GCM}‐11_{K_{j}}\left(n,m\right)\right)}^{2}}$$

where $\mathrm{GCM}-11_{K_{i}}\left(n,m\right)$ is the graphlet correlation matrix‐11's value of network *K*_*i*_ for orbits *n* and *m*.

### Assessing structural similarity using mean pairwise GCD‐11

To quantify web structural similarity, we measured and compared the mean pairwise GCD‐11 between defined sets of food webs (see Appendix S1: Section S1.3 for more information). Here, mean pairwise GCD‐11 can be thought of as a measure of structural dispersion, where the average of the pairwise GCD‐11s between all webs in a given set of webs is computed (i.e., mean of the pairwise GCD‐11s given by Equation 1). We partitioned and evaluated similarity between: (1) webs from the same ecosystem and webs from different ecosystems; (2) webs from the same publication source and webs each sourced from a different publication; and (3) aquatic webs constructed using Ecopath and aquatic webs constructed using any other method. With regard to (1), to ensure that the ecosystem groupings of “aquatic,” “aquatic and terrestrial,” and “terrestrial” were not too coarse, we also evaluated mean pairwise GCD‐11 across aquatic food webs further identified as “lake,” “marine,” “river,” “stream,” and “spring.” With regard to (2), when publications provided multiple networks, we evaluated only the mean pairwise GCD‐11 between webs from the same given publication, and when publications provided only a single network each, we evaluated the mean pairwise GCD‐11 between all webs from this group. This subset of “one food web per publication” was chosen as an imperfect null model to compare with, where the effect of publication was at least consistent between each and every web, as each web was sourced from a different publication. For visualization purposes only, the pairwise GCD‐11 between all food webs were mapped in two‐dimensional visual space using multidimensional scaling (Borg & Groenen, 2005).

We remark that no tests were performed to determine the statistical significance of differences between mean pairwise GCD‐11 values of the partitioned web groups, as our goal was to perform an exploratory data analysis rather than hypothesis testing. Moreover, as our data were unbalanced and partitioned groups had differences in the dispersion of their pairwise GCD‐11 values as well as likely differences in their centroid's location (i.e., the location of the center of the dispersion of networks for each group when projected in space), techniques like permutational multivariate ANOVA would not be useful. Furthermore, the data were pairwise dissimilarity values, and so statistical tests that use measures of variances (or standard deviations) would not have been useful without first projecting pairwise distances into an *n*‐dimensional space. We do, however, provide measures of absolute differences (i.e., differences between mean pairwise GCD‐11) to emphasize an average effect size of our measurements, which is more informative than statistical significance. We also repeated all measurements presented in the main text using median pairwise GCD‐11, to help ensure that our results were not affected by outliers (see Appendix S1: Section S1.6).

### Network size

As food web size (i.e., number of nodes) is regarded as a metric of sampling effort or intensity (Martinez et al., 1999), accounting for it may be necessary in web analyses (Brimacombe, Bodner, & Fortin, 2022). We tested to ensure neither web size nor the variability in web size influenced pairwise GCD‐11 measurements. In other words, we wanted to make sure our results were not simply an artifact of sampling effort or intensity. To do so, we compared the relationship between mean pairwise GCD‐11 and (1) food web size; and (2) variability in food web size. See Appendix S1: Section S1.7 for more information.

## RESULTS

**FIGURE 3:**
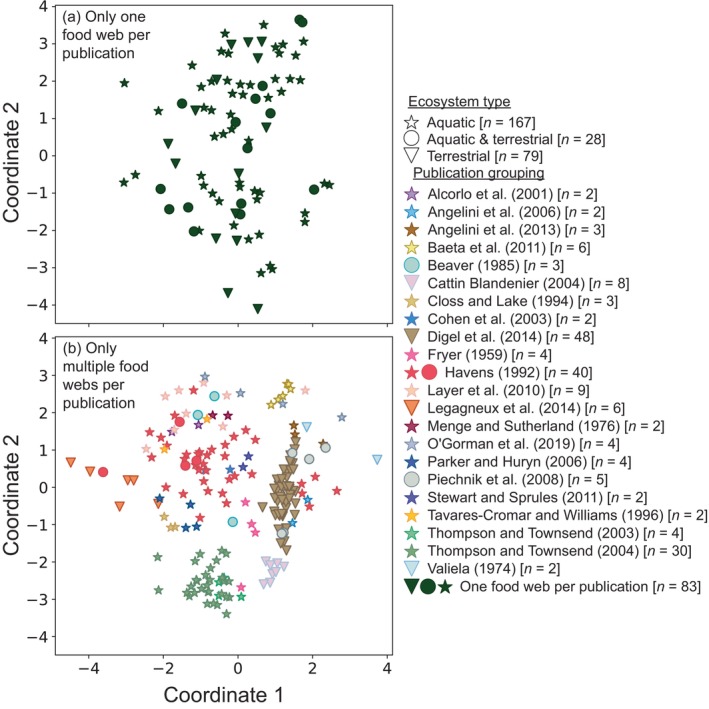
Multidimensional scaling of the pairwise graphlet correlation distance‐11 (GCD‐11) between (a) all food webs from publications that only produced a single network (*n* = 83) and (b) all food webs from publications that produced multiple networks (*n* = 191). Each symbol in the plot is a single food web, where color reflects the respective food web's source publication grouping, and shape reflects the ecosystem type each food web represents. See Appendix S1: Figure S7 for the distribution of all pairwise GCD‐11s projected here. This visual mapping is only an approximation of the high‐dimensional true pairwise GCD‐11s between all food webs.

There were no apparent differences between pairwise GCD‐11s (Figure 3) across ecosystems' food webs to suggest that specific *biological and environmental factors* associated with ecosystem type coherently influenced web structure. Food webs representing “aquatic” or “aquatic and terrestrial” were found to be comparatively structurally similar, having a mean pairwise GCD‐11 of 3.07 and 3.04, respectively (Table 1). Food webs representing “terrestrial” ecosystems were found to be more structurally similar than webs from “aquatic” and “aquatic and terrestrial,” having a mean pairwise GCD‐11 of 2.41. However, this lower mean pairwise GCD‐11 between “terrestrial” food webs was likely driven by similarities between webs sourced exclusively from Digel et al. (2014) (*n* = 48), as removing these webs increased the mean pairwise GCD‐11 of “terrestrial” food webs to 3.53. Given that the mean pairwise GCD‐11 between webs of different ecosystems were similar to webs within the same ecosystems (3.11 for “aquatic” and “aquatic and terrestrial,” 3.08 for “aquatic” and “terrestrial,” 2.96 for “aquatic and terrestrial” and “terrestrial”), ecosystem type—and hence a suite of shared biotic or abiotic drivers—appeared to have no measurable effect on food web structure. Similar patterns were also found when comparing webs from specific types of aquatic systems: “lake,” “marine,” “river,” and “stream” (Appendix S1: Section S1.5). Note that “spring” aquatic food webs were omitted from this analysis as only a single spring food web was identified and measures of structural similarity require ≥2 webs.

**TABLE 1:** Mean pairwise graphlet correlation distance‐11 (GCD‐11) between food webs sampled from the same type of ecosystem or different type of ecosystem.

	Aquatic	Aquatic and terrestrial	Terrestrial
**Aquatic**	3.07 (*n* = 167)		
**Aquatic and terrestrial**	3.11	3.04 (*n* = 28)	
**Terrestrial**	3.08	2.96	2.41 (*n* = 79)
			3.53 (*n* = 31)^a^

*Note*: Number of webs from each ecosystem are identified in parentheses. “Aquatic” food webs include those from marine, lakes, rivers, streams, and springs, “aquatic and terrestrial” food webs include those from salt marshes, ponds, bogs, mudflats, pitcher plants, and tree holes filled with water, and “terrestrial” food webs include those from sand dunes, forests, meadows, prairie, and farmlands.

^a^ After removing all *n* = 48 “terrestrial” food webs sourced from Digel et al. (2014).

**FIGURE 4:**
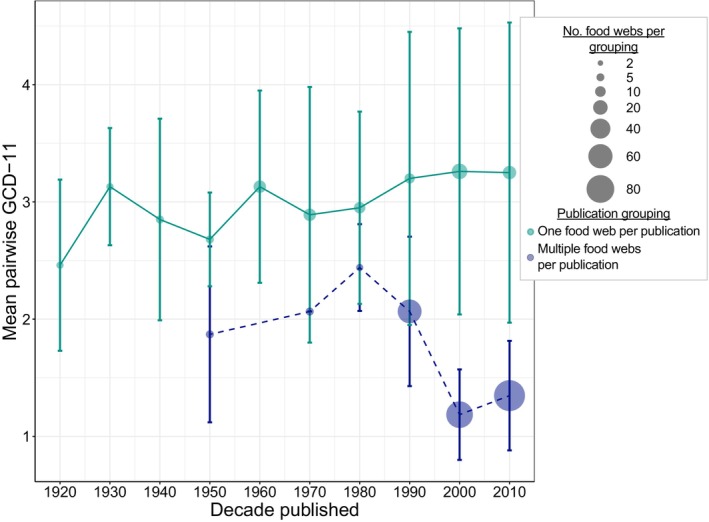
Mean pairwise graphlet correlation distance‐11 (GCD‐11) by decade of publication between food webs sourced from publications that each produced only a single network (teal solid line) and multiple food webs sourced from the same publication, weighted by the number of networks produced by each publication (blue dashed line). Circle size corresponds to the number of food webs published in each decade. Bars represent mean standard deviation of the pairwise GCD‐11 between specified subsets of food webs, which, for decades representing publications that provided multiple networks, is weighted by each publication's number of networks.

In contrast, publication source had a much stronger effect on web structure. The multiple food webs that were sourced from the same publication were on average much more structurally similar—by a factor of about two—than webs sourced from publications that each provided only a single network (i.e., mean pairwise GCD‐11: 1.51 vs. 3.13, respectively, Table 2). Recall that webs sourced from publications that each produced only a single network were used as an imperfect control to capture a possible publication effect on food web structure. Interestingly, over 85% of the structural similarities measured between food webs that shared a publication source in this study had a pairwise GCD‐11 ≤2.5. In comparison, only about 30% of the structural similarities of food webs from publications that produced only a single network and 30% of the structural similarities of all other possible pairwise distances between webs (e.g., between two food webs from two different publications that produced multiple networks) had pairwise GCD‐11 ≤2.5 (Appendix S1: Section S1.4). Moreover, the majority (i.e., about 62%) of the smallest pairwise GCD‐11s (i.e., those ≤1.5) measured across all food webs were only between those webs sourced from the same publication that produced multiple networks, despite only making up 7% of total pairwise distances. When mean pairwise GCD‐11 measures were averaged by decade, food webs sourced from publications that produced only a single network all had comparatively large mean pairwise GCD‐11 over time (i.e., >2.45; teal solid lines/points in Figure 4). However, considering only webs sourced from publications that produced multiple networks, webs from the same publication published after the 1990s were on average about 1.6 times more structurally similar than webs published before or during the 1990s (mean pairwise GCD‐11 of 1.28 vs. 2.07, respectively, Table 2, and blue dashed line/points in Figure 4).

**TABLE 2:** Mean pairwise graphlet correlation distance‐11 (GCD‐11) between food webs sourced from the same publication grouping.

Publication grouping	Mean pairwise GCD‐11	No. food webs	No. publications
One food web per publication	3.13	83	83
Multiple food webs per publication	1.51^a^	191	22
Multiple food webs per publication (before or during 1990s)	2.07^a^	56	7
Multiple food webs per publication (after 1990s)	1.28^a^	135	15

*Note*: Multiple food webs sourced from the same publication are termed “multiple food webs per publication” and food webs sourced from publications that each produced only a single network are termed “one food web per publication.” See Appendix S1: Table S6 for a list of publications that provided more than one food web and each publication's mean pairwise GCD‐11.

^a^ Calculated by taking the average of the mean pairwise GCD‐11s between food webs from the same publication, weighted by the number of networks produced by each publication.

Within aquatic food webs, there was no strong evidence to suggest that *network construction methodology* (i.e., Ecopath) coherently influenced web structure. Although the 28 aquatic webs that were constructed using Ecopath had a minimally lower mean pairwise GCD‐11 than all 167 “aquatic” food webs (2.55 vs. 3.07, respectively), this moderate difference was likely due to a publication effect, that is, four publications contributed 13 of the 28 Ecopath webs. Once we removed this publication effect, the mean pairwise GCD‐11 between aquatic webs constructed via Ecopath increased to 2.78. This structural similarity was only marginally improved compared with the mean pairwise GCD‐11 of 3.02 found between aquatic food webs not constructed via Ecopath (see Appendix S1: Section S1.8).

## DISCUSSION

Using a collection of 274 commonly reused and freely available food webs from four repositories, we found food web structure to be strongly determined by the publication source of networks. This suggests a significant lack of comparability among food webs sourced from different publications, due to cryptic publication effects embedded within the structure of all webs. Consequently, caution should be exercised when adopting food webs sourced from different publications to infer structural properties about their respective ecological communities.

Although we expect *biological and environmental factors* to have a strong influence on species interactions (e.g., Abdala‐Roberts et al., 2019; Brose et al., 2019), we found no evidence that ecosystem type coherently influenced the structure across all freely available food webs. Specifically, the structural similarity between webs from the same ecosystem (i.e., “aquatic,” “aquatic and terrestrial,” and “terrestrial”) was close to that found between webs from different ecosystems (e.g., between “aquatic” and “aquatic and terrestrial”) (Table 1). The absence of increased structural similarity among webs from the same ecosystem may be attributed to the fact that webs were built using distinctly diverse *sampling strategies* and *network construction methodologies*. In the “aquatic” ecosystem, for example, Peterson (1979) sampled the aquatic environment using transects and based feeding interactions on field observations, reports in the literature, and feeding responses in an aquarium, whereas Parker and Huryn (2006) sampled the aquatic environment using 100‐meter study reaches, and based feeding interactions on only the gut contents of caught invertebrates and a single fish species. While we recognize that our reported ecosystem type is a coarse categorization, we also did not find improved structural similarity within the more precise subsets of “aquatic” food webs identified as sampled from “lake,” “marine,” “river,” or “stream” ecosystems (Appendix S1: Section S1.5). When limiting webs to a single ecosystem type and using only those constructed with the same methodology (i.e., Ecopath), we still only observed a marginal increase in their degree of structural similarity (Appendix S1: Section S1.8). Hence, neither collections of freely available webs from the same ecosystem nor freely available webs from the same ecosystem *and* built using the same *network construction methodology* appear to substantively influence structure coherently.

From the outset, it may have been obvious that publication source would have strong influence on structure (e.g., Closs & Lake, 1994), but it was less clear that this effect would mask other drivers that we could detect across all freely available webs (Figure 3). We are not suggesting that *biological or environmental factors* do not shape food web structure. Rather, *biological or environmental factors* paired with *sampling strategies*, and *network construction methodologies* are holistically, uniquely, and cryptically captured in a publication's food web(s), leaving something like a “structural fingerprint” within each web, which make comparing networks from different publications difficult. The reason for this is made plainly evident when comparing webs sourced from Parker and Huryn (2006) and Valiela (1974), wherein the former is only concerned with the daily arthropod interactions found in bovine dung, while the latter is mainly concerned with the interactions between aquatic invertebrates and a single fish species across a month (Figure 1). In this light, it is almost trivial that webs sourced from the same publication appeared about (1) two times more structurally similar than either webs from the same ecosystem or webs each sourced from different publications (Tables 1 and 2, respectively), and (2) 1.84 times more structurally similar than aquatic webs constructed using Ecopath (Appendix S1: Section S1.8). The same strong publication effect we found here also conforms with that previously found by Brimacombe et al. (2023), where bipartite networks from the same publication were also about two times more structurally similar to each other than bipartite networks each sourced from different publications. It is important to note that while we found multiple webs sourced from the same publication to be structurally unique (i.e., had a publication's “structural fingerprint”), webs sourced from publications that each produced only a single web also have their own publication's structural fingerprint, but it could not be revealed using our methods. Like drawing a line requires at least two points, we needed at least two food webs sourced from the same publication to deduce that publication's structural fingerprint.

We are not the first to recognize the issues with reusing collections of freely available food webs. In fact, guided by the many ways food webs can be structured differently, researchers in the 1980s/1990s challenged the very utility of freely available webs as data for meaningfully testing ecological hypotheses (Dunne, 2006; Pringle & Hutchinson, 2020). Recently, studies have begun to reveal some of these previously outlined drawbacks. In particular, quantitative measurements have begun to test how differences in *sampling strategies* and *network construction methodologies* influence network structure, including the amount of area sampled (Galiana et al., 2022), amount of sampling effort (Banašek‐Richter et al., 2004; Bersier et al., 1999), and node taxon resolution (Hemprich‐Bennett et al., 2021). Altogether, these findings—along with our quantitative results—highlight the many complex drivers shaping food web structure that can make network comparison difficult. This is likely the reason why many studies that reuse collections of freely available bipartite networks built by many different researchers often do not find significant relationships in network structure across space (Brimacombe, Bodner, Michalska‐Smith, et al., 2022).

As far as we know, we are the first to have found that structural similarity between freely available food webs sourced from the same publication has generally increased across time (blue dashed line of Figure 4). This somewhat agrees with the assertion that networks published before 1990 may not have been built with the intention of evaluating structure (Carpentier et al., 2021). However, we find this true only of publications that produce multiple webs after the 1990s, and not for those webs each sourced from a unique publication (teal solid line of Figure 4). The increase in web structural similarity within a given publication may be due to the recommendations made in the late 1980s and early 1990s to improve the ways in which food webs are built (e.g., Cohen et al., 1993; Lawton, 1989; Winemiller, 1990).

The cryptic and nonlinear ways the classes of structure act holistically within a publication's own food web(s) likely make it erroneous to simply control for publication via a random effect and then deem networks comparable. As each freely available web is built by researchers for their own motives (Goldwasser & Roughgarden, 1993), the influence of each one of the three classes of structure varies substantially between publications. Hence, simply controlling for publication is unlikely to remedy the many nonlinear ways webs can be structurally different within and across publications. Importantly, it is also not possible to control for publication as a random effect in cases where food webs were each sourced from a single publication, which comprise a large portion of the freely available food webs (i.e., 83 of the 274 food webs in our study). In such scenarios, each publication grouping would contain only a single data point (i.e., food web), making it impossible to assess relationships between network structure and explanatory variables. Moreover, attempting to control for differences in the three classes that influence structure between webs is made difficult, if not impossible, by the lack of standardizations taken across publications (Borrelli et al., 2023; Cohen et al., 1993; Vázquez et al., 2022). The network metadata that would otherwise indicate how the different classes of structure influence each food web, for example, the amount of time or area used to encapsulate an ecological community as a network (i.e., *sampling strategies*), the biological evidence used to define links between nodes (i.e., *network* *construction methods*), or the type of environment the ecological community is exposed to (i.e., *biological and environmental factors*), are almost always absent (Kita et al., 2022; Poisot, Baiser, et al., 2016).

We recognize that our findings regarding structural similarity are not infallible, but we believe our conclusions regarding publication's effect on freely available food web structure are robust to variations in selected networks. First, while networks with weighted links (e.g., biomass, frequency of interaction; Cohen et al., 1993; Guimarães, 2020) have been touted as reflecting a more realistic ecological community (Banašek‐Richter et al., 2004; Bersier et al., 2002; Vázquez et al., 2022), we suspect that using weighted webs would result in similar patterns as our results with binary webs. As the litany of weighted interaction definitions may render a publication's web(s) even more unique by the chosen interaction definition, publication is likely to constrain its own webs' structure more when building weighted food webs. Second, while reducing the set of webs to only those well sampled or controlling for sampling effort has been recommended (Goldwasser & Roughgarden, 1993; Martinez et al., 1999; Winemiller, 1990), we believe that implementing this restriction is also unlikely to eliminate publication's effect on structure. As webs from the same publication have been built with similar approaches, these webs would have also been built with similar sampling effort. It follows that if webs with high sampling effort were sourced from publications that produced multiple webs, all other webs from those publications would also have high sampling effort, and thus the publication effect would still be present. Third, although we did not use directed subgraphs (i.e., graphlets) to analyze food webs as is often done, for example, Borrelli (2015) and Cirtwill and Wootton (2022), we do not believe doing so would fundamentally alter our results. We hypothesize that including direction in the edges of graphlets would reveal more structural discrepancies between publications' network(s), and thus make them more unique and difficult to compare. For example, a publication's web consisting of a single top predator would likely become apparent and identifiable from a different publication's web that has many top predators. These differences can be entirely dependent upon the goals of the researchers building the networks rather than the biology of the system itself, for example, the difference between the food webs from Parker and Huryn (2006) with a single top fish predator and Valiela (1974) with many top arthropod predators.

Looking forward, there are opportunities to improve our access to a greater number of freely available empirical food webs built by different researchers that are also less problematic to compare. The most ambitious suggestion involves a collaborative effort, in which a global set of food webs is built in a consistent and standardized manner by different researchers across the globe (Cohen et al., 1993; Winemiller, 1990). Currently, much of the structure of freely available species interaction networks is a blackbox: a result of different combinations of the drivers from the three different classes of structure applied in unbeknownst ways. Having available many food webs built using consistent and standardized protocols would allow for a more effective comparison of their structure. A more immediate and achievable remedy is for authors of food webs to include as much information about the drivers of structure that each web experiences in their metadata (Kita et al., 2022; Poisot, Stouffer, et al., 2016). As users of free data, we could then more easily decide which sets of webs are comparable or attempt to control for these differences in reported structural drivers. It is also perhaps possible to improve existing webs using inferential methods. While these sorts of approaches are novel, they may be able to overcome sampling bias and data deficiency issues that plague species interaction networks by predicting interactions in cases where no such interaction has been recorded using data from other networks (Poisot et al., 2023). Of course, these methods still require validation to determine whether predicted interactions are plausible.

## CONCLUSION

In our study, we demonstrate that the structure of food webs is primarily defined by each web's publication source. This strong publication effect likely arises as webs are exposed to their publication's distinct combinations of structural drivers that can be broadly categorized into three classes: *biological and environmental factors*, *sampling strategies*, and *network construction methods*. Unfortunately, simply controlling for the publication source of each web is insufficient when comparing webs sourced from different publications, as the *real* structural drivers (i.e., of the three aforementioned classes) are likely holistically acting on the structure of webs in nonlinear ways. We suggest that one of the simplest approaches to improve web comparability is for builders of a publication's web(s) to report in metadata the different ways the three classes of structure influence each food web. In this way, researchers who adopt freely available webs can attempt to control for the nonlinear and interacting ways in which the different structural drivers may act.

## AUTHOR CONTRIBUTIONS

Chris Brimacombe and Korryn Bodner conceived the study with help from Dominique Gravel, Shawn J. Leroux, Timothée Poisot, and Marie‐Josée Fortin. Chris Brimacombe and Korryn Bodner collected the networks and analyzed the data. Chris Brimacombe and Korryn Bodner wrote the manuscript, and all authors made contributions to subsequent revisions.

## CONFLICT OF INTEREST STATEMENT

The authors declare no conflicts of interest.

## Supporting information


Appendix S1.

## Data Availability

Data and code (Chrisb590, 2024) are available in Zenodo at https://doi.org/10.5281/zenodo.13769533.
