# Supplementary material for: Publication‐driven consistency in food web structures: Implications for comparative ecology
Source: Ecology. 2024 Nov 21;106(1):e4467. doi: 10.1002/ecy.4467 (PMC11732785; doi:10.1002/ecy.4467)
Supplement: Supplementary file 1 — Appendix S1. [file ECY-106-e4467-s001.pdf]

Publication-driven consistency in food web structures: Implications for comparative ecology

Ecology

Chris Brimacombe, Korryn Bodner, Dominique Gravel, Shawn J. Leroux, Timothée Poisot, Marie-Josée Fortin

Appendix S1

Section S1.1 Errors in food webs

Here, we provide the changes we made to the collected binary webs' adjacency matrices (Table S1), where rows and columns correspond to biological entities.

Table S1: Changes to food web adjacency matrices. Although not listed here, it is important to ensure no extra white space characters are included either in front or behind column/row string names, otherwise R (R Core Team, 2023) will interpret these names as different nodes if there are also rows/columns with the same string name but without additional white space characters.

Network name	Changes made	Network result
WEB33_	Row labelled "Gyraulus costulatus\" was merged with the already present row labelled "Gyraulus costulatus".	Reduced network size by 1 node.
WEB41_	2 rows and 2 columns labelled "tuna" (different interaction configurations for each row and each column) were merged into a single row and a single column, respectively.	
WEB214_	Row labelled "Eukieffidriella 'naonella' type" was merged with the already present row labelled "Eukiefferiella 'naonella' type".	Reduced network size by 1 node.
	2 rows labelled "Melosira italica" (different interaction configurations for each row) were merged into a single row.	
WEB215_	Row labelled "Eukieferiella pseudomontana" was merged with the already present row labelled "Eukiefferiella pseudomontana".	Reduced network size by 1 node.
WEB217_	Row labelled "Eukieffidrella pseudomontana" was merged with the already present row labelled "Eukiefferiella pseudomontana".	Reduced network size by 1 node.
	Row labelled "Fatigia pele" was merged with the already present row labelled "Fattigia pele".	Reduced network size by 1 node.
WEB218_	Row labelled "Austrosimulium austranse" was merged with the already present row labelled "Austrosimulium australense".	Reduced network size by 1 node.
	Row labelled "Eukiefidrella brundini" was merged with the already present row labelled "Eukiefferiella brundini".	Reduced network size by 1 node.

Table continued …

. . . Continuation of Table S1.

Network name	Changes made	Network result
	Row labelled “Eukieflidrella brundini” was merged with the already present row labelled “Eukiefferiella brundini”.	Reduced network size by 1 node.
WEB219_	Row labelled “Pycnocntria evecta” was merged with the already present row labelled “Pycnocentria evecta”.	Reduced network size by 1 node.
	Row labelled “Zelanoperla sp.” was merged with the already present row labelled “Zelandoperla sp.”.	Reduced network size by 1 node.
WEB220_	Row labelled “Eukieflidriella brundini” was merged with the already present row labelled “Eukiefferiella brundini”.	Reduced network size by 1 node.
	Row labelled “Eukieffidriella brundini” was merged with the already present row labelled “Eukiefferiella brundini”.	Reduced network size by 1 node.
	Row labelled “Tiphobiosis montana” was merged with the already present row labelled “Tiphobiosis montana”.	Reduced network size by 1 node.
	Row labelled “Aphrophila neozelandicus” was merged with the already present row labelled “Aphrophila neozelandica”.	Reduced network size by 1 node.
WEB221_	Row labelled “Aotepsyche” was merged with the already present row labelled “Aoteapsyche”.	Reduced network size by 1 node.
WEB223_	Row labelled “Cymbella kappi” was merged with the already present row labelled “Cymbella kappii”.	Reduced network size by 1 node.
WEB224_	Row labelled “Stictoclcaius” was merged with the already present row labelled “Stictocladius”.	Reduced network size by 1 node.
WEB236_	2 rows labelled “Zelandoperla agnetis (McLellan)” (different interaction configurations for each row) were merged into a single row.	
	Row labelled “Eukiefidrella” was merged with the already present row labelled “Eukiefferiella”.	Reduced network size by 1 node.
	Row labelled “Eukiefiriella” was merged with the already present row labelled “Eukiefferiella”.	Reduced network size by 1 node.
	Row labelled “Stictoclcaius” was merged with the already present row labelled “Stictocladius”.	Reduced network size by 1 node.
	2 columns labelled “Zelandoperla agnetis (McLellan)” (different interaction configurations for each column) were merged into a single column.	
WEB238_	2 rows labelled “Achnanthes linearis” (different interaction configurations for each row) were merged into a single row.	

Table continued . . .

…Continuation of Table S1.

Network name	Changes made	Network result
WEB240_	2 rows labelled "Achnanthes linearis" (different interaction configurations for each row) were merged into a single row.	
WEB244_	2 rows labelled "Achnanthes linearis" (different interaction configurations for each row) were merged into a single row.	
WEB246_	2 rows labelled "Achnanthes linearis" (different interaction configurations for each row) were merged into a single row.	
WEB257_	Row labelled "Amphithoe valida" was merged with the already present row labelled "Ampithoe valida".	Reduced network size by 1 node.
WEB258_	Row labelled "Amphithoe valida" was merged with the already present row labelled "Ampithoe valida".	Reduced network size by 1 node.
WEB259_	Row labelled "Amphithoe valida" was merged with the already present row labelled "Ampithoe valida".	Reduced network size by 1 node.
WEB260_	Row labelled "Amphithoe valida" was merged with the already present row labelled "Ampithoe valida".	Reduced network size by 1 node.
WEB262_	Row labelled "Glicera tridactyla" was merged with the already present row labelled "Glycera tridactyla".	Reduced network size by 1 node.
WEB320_	Rows labelled "Import", "Sum", and "(1-Sum)" were removed.	Reduced network size by 3 nodes.
WEB321_	Rows labelled "Import", "Sum", and "(1-Sum)" were removed.	Reduced network size by 3 nodes.
WEB322_	Rows labelled "Import", "Sum", and "(1-Sum)" were removed.	Reduced network size by 3 nodes.
WEB323_	Row labelled "Import" was removed.	Reduced network size by 1 node.
WEB324_	Rows labelled "Import", "Sum", and "(1-Sum)" were removed.	Reduced network size by 3 nodes.
WEB338_	Rows labelled "Import" and "Discard" were removed.	Reduced network size by 2 nodes.
WEB345_	2 rows and 2 columns labelled "Medium-sized ciliates (herbivore)" (different interaction configurations for each row and each column) were merged into a single row and a single column, respectively.	
WEB348_	Row labelled "Polychates" was merged with the already present row labelled "Polychaete".	Reduced network size by 1 node.
WEB350_	Row labelled "Polychates" was merged with the already present row labelled "Polychaetes".	Reduced network size by 1 node.
	2 rows labelled "Ostracods" (different interaction configurations for each row) were merged into a single row.	

Table continued …

. . . Continuation of Table S1.

Network name	Changes made	Network result
WEB352_	Row labelled "Import" was removed.	Reduced network size by 1 node.
WEB353_	Row labelled "Import" was removed.	Reduced network size by 1 node.
WEB359_	11 rows and 11 columns labelled "unknown bacterium" (different interaction configurations for each row and column) were merged into a single row and a single column, respectively.	
	12 rows and 12 columns labelled "unknown protozoan" (different interaction configurations for each row and column) were merged into a single row and a single column, respectively.	
	3 rows and 3 columns labelled "Sphingomonas" (different interaction configurations for each row and column) were merged into a single row and a single column, respectively.	
	3 rows and 3 columns labelled "Flectobacillus" (different interaction configurations for each row and column) were merged into a single row and a single column, respectively.	
	2 rows and 2 columns labelled "Chromobacterium" (different interaction configurations for each row and column) were merged into a single row and a single column, respectively.	
Carpinteria_	2 rows and 2 columns labelled "eugregarine" (different interaction configurations for each row and each column) were merged into a single row and a single column, respectively.	
Beaver_Lake_	Row and column labelled "Salmo rutta" was corrected to "Salmo trutta".	
Kongsfjorde_	Row labelled "Eumicotremus derjugini" was merged with the already present column labelled "Eumicrotremus derjugini".	Reduced network size by 1 node.
mown_Clmown1_	Changed row and column labelled "Edaphus_blÅ¸hweissi" to "Edaphus".	
mown_Scmown2_	Changed row and column labelled "Edaphus_blÅ¸hweissi" to "Edaphus".	
not_mown_ClControl1_	Changed row and column labelled "Edaphus_blÅ¸hweissi" to "Edaphus".	
not_mown_ScControl1_	Changed row and column labelled "Edaphus_blÅ¸hweissi" to "Edaphus".	
not_mown_ScControl2_	Changed row and column labelled "Edaphus_blÅ¸hweissi" to "Edaphus".	

Section S1.2 Graphlet correlation distance-11 example

Given a food web, such as "Network 1" depicted in Figure S2, we first count the number of times nodes occupy orbit positions (Figure S1). Specifically, the number of times nodes occupy the orbits of graphlets $\mathbb{G}_1$ (orbit 1), $\mathbb{G}_2$ (orbits 2 and 3), $\mathbb{G}_3$ (orbits 4 and 5), $\mathbb{G}_4$ (orbits 6 and 7), $\mathbb{G}_5$ (orbit 8), and $\mathbb{G}_6$ (orbits 9, 10, and 11). In tallying the number of times a node occupies different orbit positions, the *graphlet degree vector-11* for a node is constructed. In Figure S2, we provide the graphlet degree vector-11 for "node A" of Network 1.

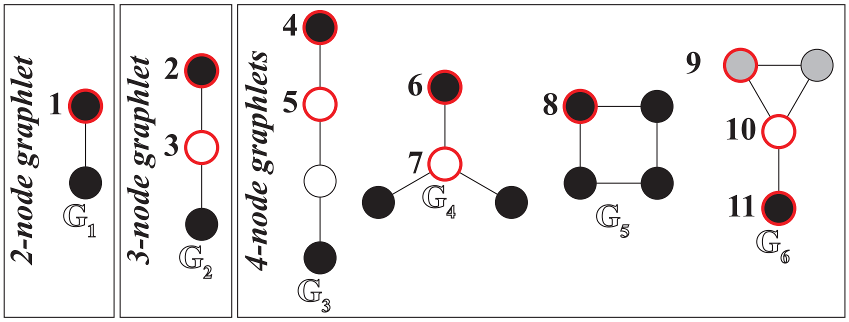

Figure S1: The six graphlets ($\mathbb{G}_i$) consisting of two-to-four nodes, and their respective orbits (i.e., the corresponding 11 numerically labelled node positions). Each unique shade in a single graphlet corresponds to a unique orbit in that graphlet.

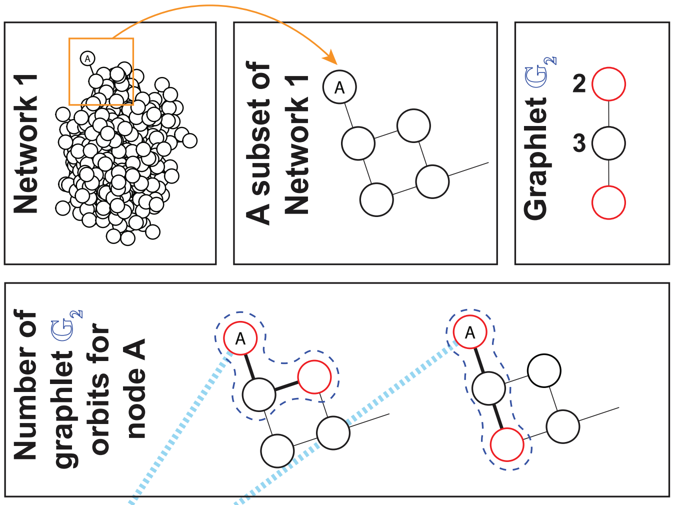

Orbit	1	2	3	4	5	6	7	8	9	10	11
Node A	1	2	0	2	0	1	0	0	0	0	0

Graphlet degree vector-11

Figure S2: Example calculation of the counts for orbit 2 in a graphlet degree vector-11 for node A of Network 1.

Once graphlet degree vector-11s for each node in a food web are determined, the *graphlet correlation matrix-11* can be assembled. In doing so, all possible Spearman's correlations be-

tween the number of times all nodes in a food web occupy specific orbits are evaluated (see the highlighted green boxes in Figure S3 as an example of a single Spearman's correlation for Network 1).

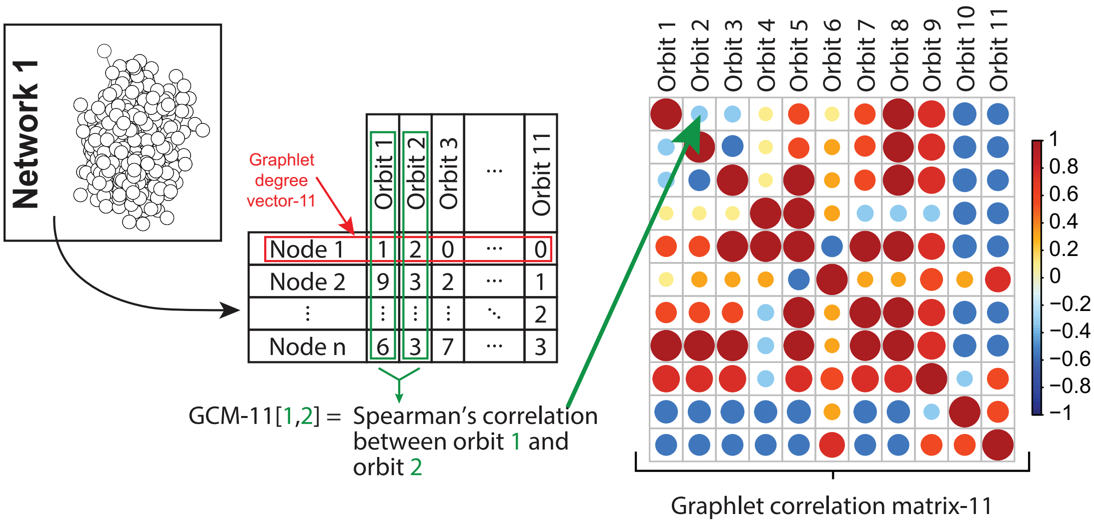

Figure S3: Example calculation of a graphlet correlation matrix-11 (GCM-11) for Network 1 using the 11 orbits that comprise the graphlet correlation distance-11 method. First, graphlet degree vector-11s for each node in the network are calculated (a single vector is highlighted in red). Next, Spearman's correlations are calculated between all pairs of orbits using the number of times each node occupies each orbit (an example of the vectors used in a single correlation is highlighted in green). The resulting correlations form entries within the GCM-11.

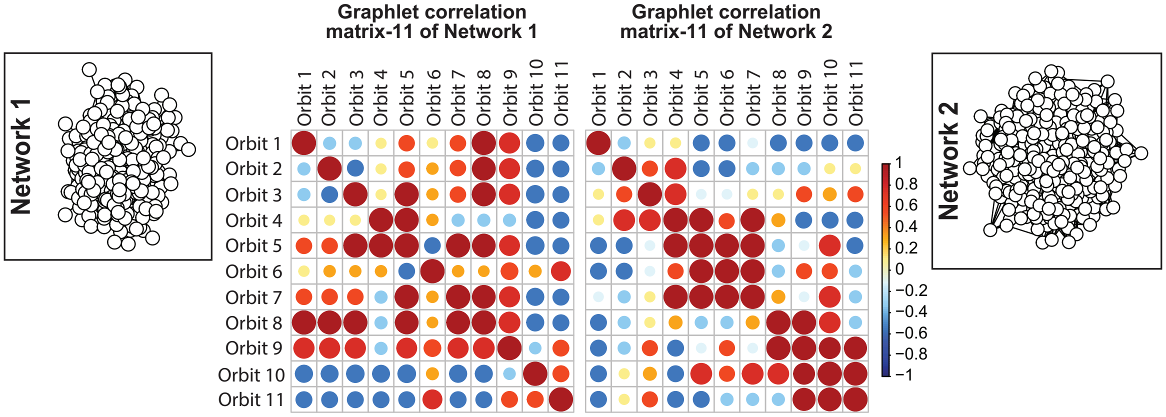

Pairwise Euclidean distance

$$\text{GCD-11}\,(K_i, K_j) = \sqrt{\sum_{n=1}^{11}\sum_{m=n+1}^{11}\left(\text{GCM-11}_{K_i}(n,m) - \text{GCM-11}_{K_j}(n,m)\right)^2}$$

$$= \sqrt{(-0.24 - [-0.19])^2 + \cdots + (0.94 - 0.58)^2}$$

$$= 2.3$$

Figure S4: The formula and an example calculation of the pairwise graphlet correlation distance-11 (pairwise GCD-11) using the two graphlet correlation matrix-11s of Network 1 and Network 2.

By computing the pairwise Euclidean distances between graphlet correlation matrix-11s, we can obtain an estimate of the topological differences between food webs in a given set. An

example of a single pairwise Euclidean distance between two graphlet correlation matrices (i.e., of two networks or food webs) is shown in Figure S4.

Using all pairwise Euclidean distances between food webs, we can visualize their dissimilarity by projecting their distances using multidimensional scaling (MDS; see Figure S5).

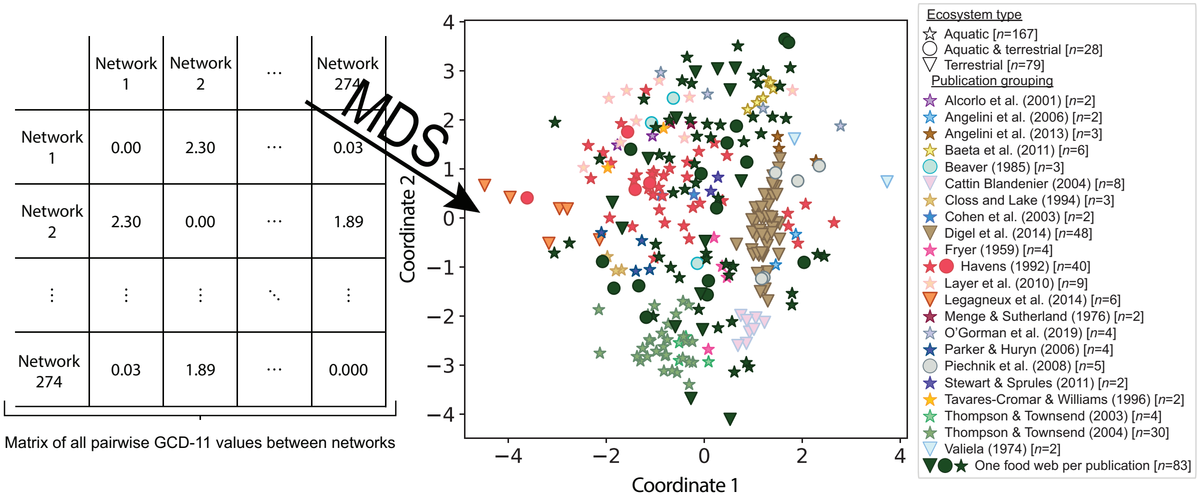

Figure S5: Multidimensional scaling (MDS) projection of all pairwise GCD-11s between the 274 food webs, as also shown in Figure 3.

Section S1.3 Example of mean pairwise GCD-11 as a dispersion metric

In Figure S6, we provide a toy example of how the mean pairwise GCD-11 between defined sets of food webs (i.e., green circles and purple squares) can be used as a metric of dispersion within those sets of webs. We note that the distances represented in the multidimensional scaling plot of Figure S6 are only a 2-dimensional best approximation to the true pairwise distances between all 7 food webs using pairwise GCD-11s (as calculated via Eq. 1). We used the “true” pairwise GCD-11s between food webs (i.e., the pairwise input data used to perform a multidimensional scaling) throughout the manuscript for all quantitative measurements.

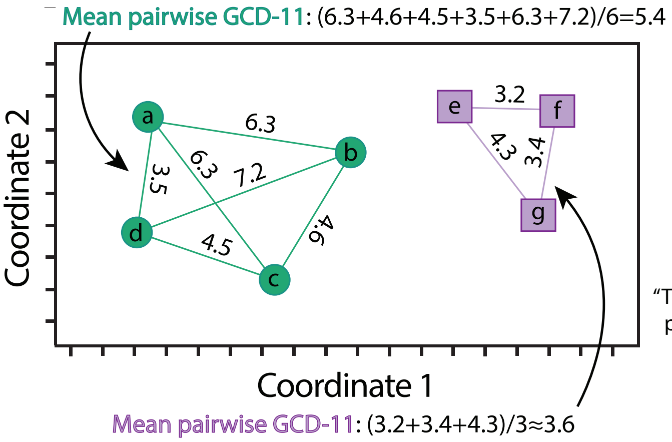

	a	b	c	d	e	f	g
a	0	6.3	6.3	3.5	9.6	12.8	12.5
b	6.3	0	4.6	7.2	3.6	6.6	6.2
c	6.3	4.6	0	4.5	7.8	10.3	8.5
d	3.5	7.2	4.5	0	10.8	13.8	12.7
e	9.6	3.6	7.8	10.8	0	3.2	4.3
f	12.8	6.6	10.3	13.8	3.2	0	3.4
g	12.5	6.2	8.5	12.7	4.3	3.4	0

“True” pairwise GCD-11s between all 7 food webs prior to visualizing in multidimensional scaling

Figure S6: Example multidimensional scaling (MDS) [plot on left] of all pairwise graphlet correlation distance-11s (GCD-11) [matrix on right] between food webs ($n = 7$) mapped in 2-dimensional space. Lines are drawn on the MDS to convey the pairwise distances between symbols/webs (but distances are obtained from the matrix). Each symbol in the plot is a single food web, where colour and shape reflects the respective food web’s grouping. Distances between webs of opposite groupings are not drawn on plot for the sake of simplicity.

Section S1.4 Distribution of all pairwise GCD-11 values

In Figure S7, we provide the distribution of all $n = 37401$ pairwise GCD-11 values between the 274 food webs used in this study, where total pairwise distances are defined by

$$\frac{\text{number of networks} \cdot (\text{number of networks} - 1)}{2} = \frac{274 \cdot 273}{2} = 37401\,. \qquad \text{(S1)}$$

The total number of pairwise distances between: (*i*) the 83 food webs sourced from a publication that produced only a single network was $n = 3403$, (*ii*) the 191 food webs sourced from 22 publications that each produced multiple networks was $n = 2487$, and (*iii*) all other webs (i.e., between two food webs sourced from two different publications that produced multiple networks *or* a food web from a publication that produced only a single network and a food web from a publication that produced multiple networks) was $n = 31511$.

Strikingly, the low pairwise GCD-11s between food webs analyzed in our study were dominated by those webs that shared a publication source. In particular, the majority (i.e., about 62%) of the smallest pairwise GCD-11s (i.e., those ≤ 1.5) measured between all food webs were only between those webs sourced from the same publication that produced multiple networks, despite only making up 7% of the total pairwise distances (i.e., 2487/37401). Moreover, 87% of all pairwise GCD-11s recorded between food webs that shared a publication source were ≤ 2.5. In comparison, only about 30% and 27% of food webs from publications that produced only a single network, and all other pairwise distances between webs, were ≤ 2.5.

These results show that it would be extremely difficult—if not impossible—to categorize food webs using the same number of webs ($n = 191$) to maximize structural similarity, other than by the publication groupings presented in this manuscript (i.e., food webs sourced from the same publication that produced multiple networks). Given that the majority of small pairwise GCD-11s are already categorized as food webs that share a publication source, there are very few pairwise GCD-11s that remain which could be categorized to further maximize structural similarity. Moreover, these categorizations would need to be based on a priori rules rooted in ecological theory, rather than categorizing food webs simply based on small GCD-11s.

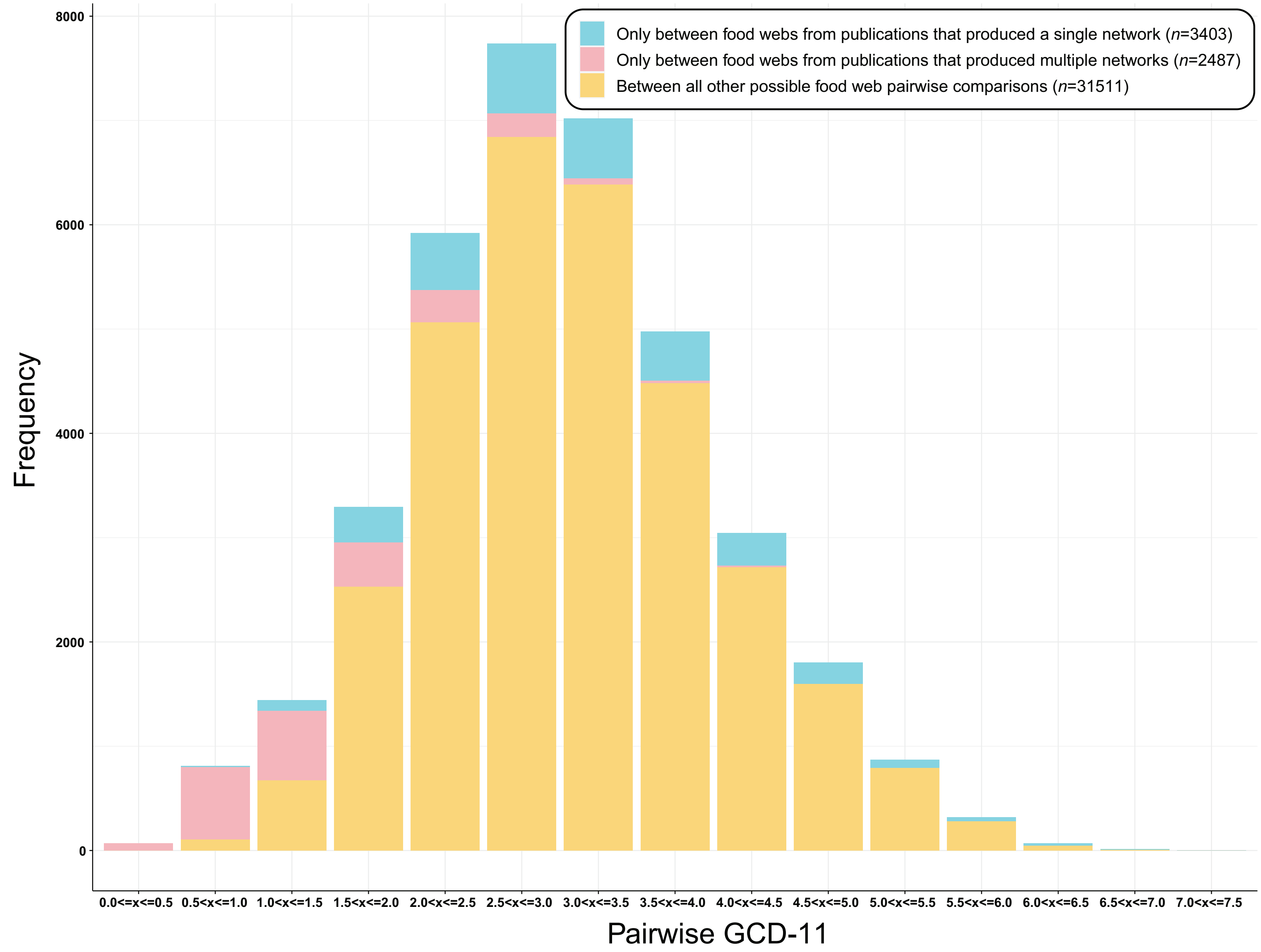

Figure S7: The distribution of all pairwise graphlet correlation distance-11s (GCD-11s) between 247 food webs. n in legend corresponds to the number of pairwise GCD-11s for each category, where all total pairwise distances is $n = 37401$ $\left[\text{i.e., } \frac{\text{number of networks}\cdot(\text{number of networks}-1)}{2} = \frac{274\cdot 273}{2}\right]$.

Section S1.5 No substantial evidence of increased structurally similarity between the "aquatic" food webs of "lake", "marine", "river", and "stream"

Here, we test whether "aquatic" food webs—when further identified down to either "lake", "marine", "river", and "stream"—have increased structurally similarity (i.e., smaller mean pairwise GCD-11). Note: we omitted the single food web sampled from a "spring" ecosystem (i.e., WEB45_) in this analysis, since it is impossible to evaluate mean pairwise GCD-11 with only a single network.

Analysis Since food webs sourced from the same publication are already known to be highly similar to each other (i.e., mean pairwise GCD-11 of 1.51 [Table 2]), we removed the publication effect from this analysis by only using a single web from a publication that provided multiple networks. As a means of reducing the burden of sampling across all possible different and unique combinations of taking a single web from each of the publications that provided multiple networks for the "aquatic" food web ecosystem, we simply randomly chose a single web from each of these publication. We refer to a collated combination of randomly chosen webs (each one from a unique publication source that provided multiple networks), and all webs sourced from publications that each provided only a single network, as a single realization. When analyzing the mean pairwise GCD-11 between food webs from the same "aquatic" ecosystem (e.g., "lake"), we averaged all mean pairwise GCD-11 values across 200 realizations between webs only from the same "aquatic" ecosystem. When analyzing the mean pairwise GCD-11 between food webs across "aquatic" ecosystems (e.g., "lake" and "marine"), we averaged all mean pairwise GCD-11 values across 200 realizations between food webs only from the two *different* "aquatic" ecosystems. Below we list the number of food webs in a realization from each of these "aquatic" ecosystems.

Data Of the "lake" food webs available from our dataset, 7 webs were sourced from publications that each provided only a single network, while 49 webs were sourced from 6 publications that each provided multiple networks (see Table S7 for list). Specifically, 3 webs were sourced from Angelini et al. (2013), 2 webs were sourced from Stewart and Sprules (2011), 2 webs were sourced from Alcorlo et al. (2001), 2 webs were sourced from Cohen et al. (2003), 4 webs were sourced from Fryer (1959), and 36 webs were sourced from Havens (1992). A given realization then consisted of 13 "lake" food webs.

Of the "marine" food webs available from our dataset, 27 webs were sourced from publications that each provided only a single network, while 8 webs were sourced from 2 publications that each provided multiple networks (see Table S7 for list). Specifically, 6 webs were sourced from Baeta et al. (2011), and 2 webs were sourced from Menge and Sutherland (1976). A given realization then consisted of 29 "marine" food webs.

Of the "river" food webs available from our dataset, 6 webs were sourced from publications that each provided only a single network, while 2 webs were sourced from 1 publication that provided multiple networks (see Table S7 for list). Specifically, 2 webs were sourced from Angelini et al. (2006). A given realization then consisted of 7 "river" food webs.

Of the "stream" food webs available from our dataset, 11 webs were sourced from publications that each provided only a single network, while 56 webs were sourced from 7 publica-

tion that provided multiple networks (see Table S7 for list). Specifically, 3 webs were sourced from Closs and Lake (1994), 9 webs were sourced from Layer et al. (2010), 4 webs were sourced from O'Gorman et al. (2019), 4 webs were sourced from Parker and Huryn (2006), 2 webs were sourced from Tavares-Cromar and Williams (1996), 4 webs were sourced from Thompson and Townsend (2003), and 30 webs were sourced from Thompson and Townsend (2004). A given realization then consisted of 18 "stream" food webs.

Table S2: Mean pairwise graphlet correlation distance-11 (GCD-11) between food webs sampled from the same type or different "aquatic" ecosystem. Number of webs from each "aquatic" ecosystem are identified in parentheses.

	Lake	Marine	River	Stream
Lake	2.58 ($n = 13$)			
Marine	2.96	3.16 ($n = 29$)		
River	2.71	3.17	2.87 ($n = 7$)	
Stream	2.93	3.24	2.97	3.21 ($n = 18$)

Findings Altogether, there is no substantial evidence that "aquatic" food webs further identified to "lake", "marine", "river", or "stream" are more structurally similar (i.e., smaller mean pairwise GCD-11 between webs from the same "aquatic" ecosystem) [Table S2]. While "lake" food webs were moderately more structurally similar to each other (i.e., mean pairwise GCD-11: 2.58)—as compared to any other non-publication type grouping studied for this paper—food webs sourced from the same publication were much more structurally similar to each other (i.e., mean pairwise GCD-11: 1.51), especially those published after the 1990s (i.e., mean pairwise GCD-11: 1.28) [Table 2]. Moreover, the other three types of "aquatic" food webs had considerably larger mean pairwise GCD-11 than "lakes" (i.e., "marine": 3.16, "river": 2.87, and "stream" 3.21).

Section S1.6 Median pairwise GCD-11

Table S3: Median pairwise graphlet correlation distance-11 (GCD-11) between food webs sampled from the same type of ecosystem or different type of ecosystem. Number of webs from each ecosystem are identified in parentheses. "Aquatic" food webs include those from marine, lakes, rivers, streams, and springs, "aquatic and terrestrial" food webs include those from salt marshes, ponds, bogs, mudflats, pitcher plants, and tree holes filled with water, and "terrestrial" food webs include those from sand dunes, forests, meadows, prairie, and farmlands.

	Aquatic	Aquatic and terrestrial	Terrestrial
Aquatic	3.01 ($n = 167$)		
Aquatic and terrestrial	3.06	3.09 ($n = 28$)	
Terrestrial	3.00	2.86	2.33 ($n = 79$)<br>3.72 ($n = 31$)†

† After removing all $n = 48$ "terrestrial" food webs sourced from Digel et al. (2014).

Table S4: Median pairwise graphlet correlation distance-11 (GCD-11) between food webs sourced from the same publication grouping. Multiple food webs sourced from the same publication are termed "multiple food webs per publication" and food webs sourced from publications that each produced only a single network are termed "one food web per publication". See Table S6 for a list of publications that provided more than one food web and each publication's mean pairwise GCD-11.

Publication grouping	Median pairwise GCD-11	Number of food webs	Number of publications
One food web per publication	3.02	83	83
Multiple food webs per publication	1.15‡	191	22
Multiple food webs per publication (before or during 1990s)	2.00‡	56	7
Multiple food webs per publication (after 1990s)	1.08‡	135	15

‡ Calculated by taking the median of the median pairwise GCD-11s between food webs from the same publication.

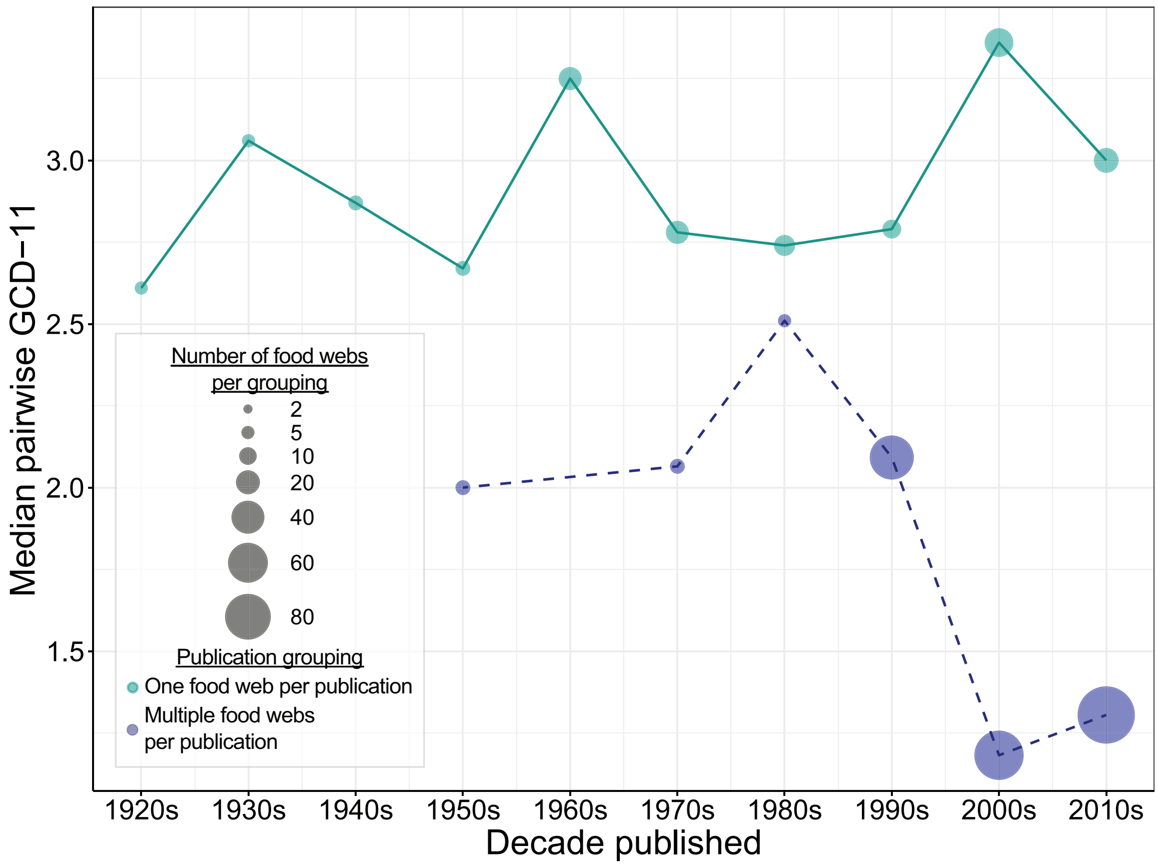

Figure S8: Median pairwise graphlet correlation distance-11 (GCD-11) by decade published between food webs sourced from publications that each produced only a single network (teal solid line) and, multiple food webs sourced from the same publication, weighted by the number of networks produced by each publication (blue dashed line). Circle size corresponds to the number of food webs published in the decade.

Section S1.7 No evidence that the number of nodes or standard deviation in the number of nodes influences pairwise GCD-11

Here, we test whether the number of nodes (i.e., web size) or standard deviation in the number of nodes (i.e., standard deviation of web size) in food webs influenced both mean and individual pairwise GCD-11.

Mean pairwise GCD-11 between food webs from publications that produced only a single network We divided webs sourced from publications that each provided only a single network into quartiles based on their number of nodes (Table S5). With respect to these quartiles (labelled Q1, Q2, Q3, and Q4), we found that although both web size (mean number of nodes for Q1: 12.52, Q2: 19.57, Q3: 28.89, and Q4: 117.95) and standard deviation in web size (mean standard deviation of nodes for Q1: 1.59, Q2: 3.09, Q3: 3.05, and Q4: 108.76) differed greatly between the quartiles, there were no large differences between their respective mean pairwise GCD-11 (mean pairwise GCD-11 for Q1: 2.70, Q2: 2.90, Q3: 3.04, and Q4: 3.05). Hence, neither web size or standard deviation in web size influenced mean pairwise GCD-11.

Mean pairwise GCD-11 between food webs from publications that produced multiple networks To keep publication a factor rather than separate webs into quartiles as was done above for webs sourced from publications that each provided only a single network, we chose to instead evaluate the regressions of both mean web size and standard deviation in web size across publications to explain mean pairwise GCD-11 (Figure S9). In both cases, neither web size nor the standard deviation in web size explained mean pairwise GCD-11 across publication ($p > 0.34$, and $p > 0.55$, respectively).

Pairwise GCD-11 between all food webs Regardless of publication source, we compared all pairwise graphlet correlation distance-11s (GCD-11s) between the 274 food webs in this study. This amounts to a total of 37401 pairwise distances:

$$\frac{\text{number of networks} \cdot (\text{number of networks} - 1)}{2} = \frac{274 \cdot 273}{2} = 37401\,. \qquad \text{(S2)}$$

In both cases, neither web size ($R^2 = 0.02$) nor standard deviation in web size ($R^2 = 0.02$) meaningfully explained pairwise GCD-11s (Figure S10).

Table S5: Mean pairwise graphlet correlation distance-11 (GCD-11) between food webs sourced from publications that each produced only a single network (i.e., one food per publication) when partitioned into quartiles based the number of nodes.

Quartile number of nodes	Mean pairwise GCD-11	Mean number of nodes	S.D. number of nodes	Number of food webs
1	2.70	12.52	1.59	23
2	2.90	19.57	3.09	21
3	3.04	28.89	3.05	19
4	3.05	117.95	108.76	20

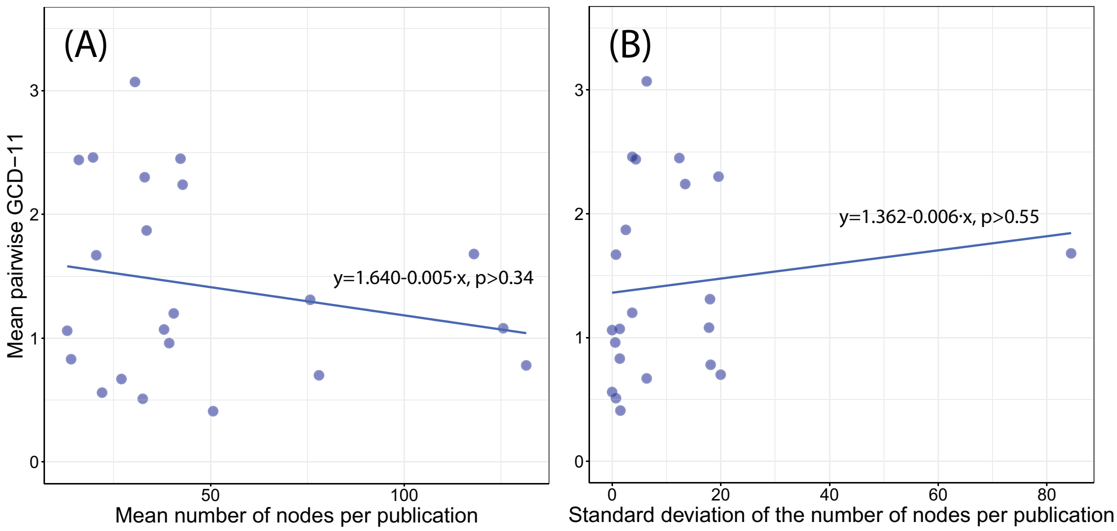

Figure S9: **(A)** Mean pairwise graphlet correlation distance-11 (GCD-11) as a function of the mean number of nodes between food webs sourced from the same publication ($n = 22$). **(B)** Mean pairwise GCD-11 as a function of the standard deviation in the number of nodes between food webs sourced from the same publication ($n = 22$). See Table S6 for exact values for the mean pairwise GCD-11, the mean number of nodes, and the standard deviation in the number of nodes between food webs sourced from the same publication.

Table S6: Mean pairwise graphlet correlation distance-11 (GCD-11) between food webs from the same publication grouping. Each food web sourced from a publication that produced only a single network belong to the grouping "one food web per publication", while multiple food webs sourced from the same publication belong to that publication's grouping.

Publication grouping	Mean pairwise GCD-11	Mean number of nodes	S.D. number of nodes	Number of food webs
Closs and Lake (1994)	0.41	50.67	1.53	3
Angelini et al. (2006)	0.51	32.50	0.71	2
Stewart and Sprules (2011)	0.56	22.00	0.00	2
Baeta et al. (2011)	0.67	27.00	6.36	6
Thompson and Townsend (2003)	0.70	78.00	19.98	4
Cattin Blandenier (2004)	0.78	131.50	18.13	8
Alcorlo et al. (2001)	0.83	14.00	1.41	2
Angelini et al. (2013)	0.96	39.33	0.58	3
Menge and Sutherland (1976)	1.06	13.00	0.00	2
Tavares-Cromar and Williams (1996)	1.07	38.00	1.41	2
Digel et al. (2014)	1.08	125.56	17.83	48
Parker and Huryn (2006)	1.20	40.50	3.70	4
Thompson and Townsend (2004)	1.31	75.73	18.01	30
Cohen et al. (2003)	1.67	20.50	0.71	2
Piechnik et al. (2008)	1.68	118.00	84.46	5
Fryer (1959)	1.87	33.50	2.52	4
Havens (1992)	2.24	42.80	13.45	40
Layer et al. (2010)	2.30	33.00	19.58	9
Beaver (1985)	2.44	16.00	4.36	3
O'Gorman et al. (2019)	2.45	42.25	12.37	4
Legagneux et al. (2014)	2.46	19.67	3.72	6
Valiela (1974)	3.07	30.50	6.36	2
One food web per publication	3.13	43.46	67.55	83

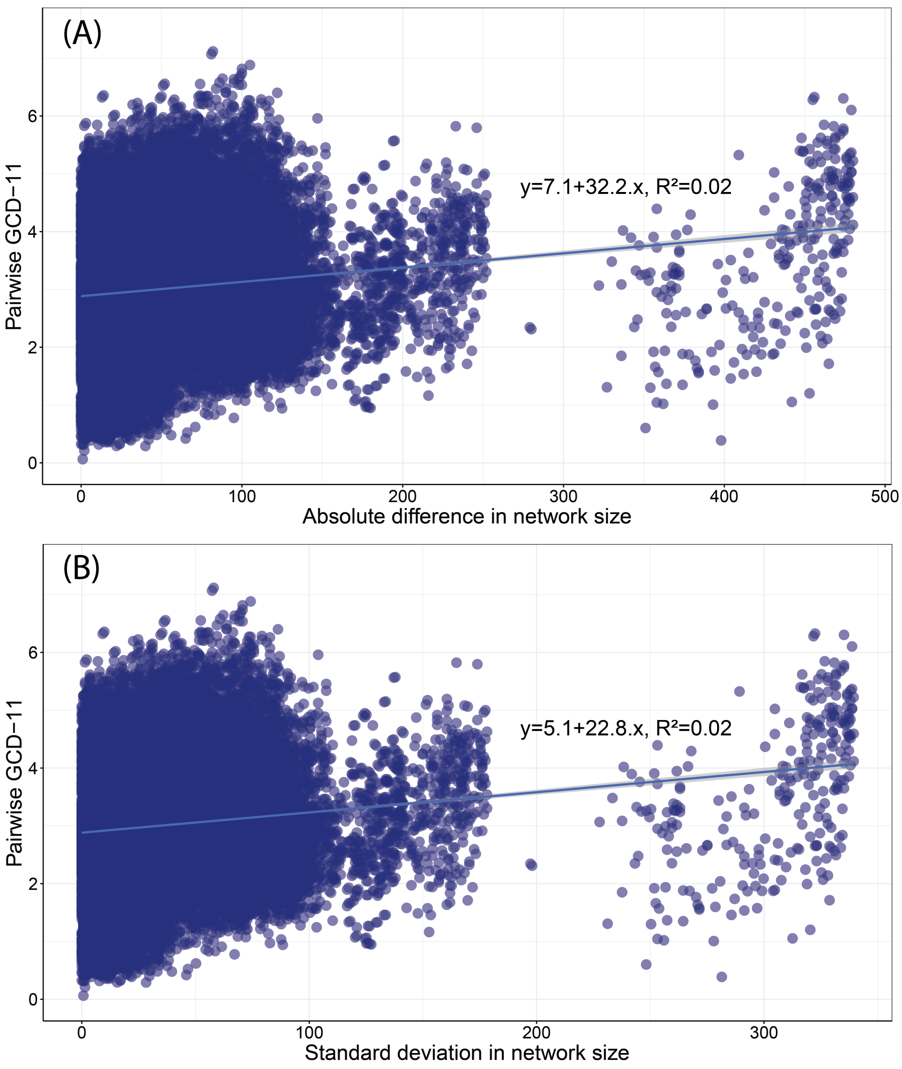

Figure S10: **(A)** All possible $n = 37401$ pairwise graphlet correlation distance-11s (GCD-11s) between the 274 food webs as a function of the absolute difference in network size (i.e., number of nodes), where each point is a pairwise GCD-11 measure between two webs. **(B)** All possible $n = 37401$ pairwise graphlet correlation distance-11s (GCD-11s) between the 274 food webs as a function of the standard deviation in network size (i.e., number of nodes), where each point is a pairwise GCD-11 measure between two webs.

Section S1.8 No substantial evidence "aquatic" food webs constructed using Ecopath are more structurally similar than "aquatic" food webs not constructed using Ecopath

Overall, there is no substantial improvement in structural similarity when food webs were built using similar *network construction methodology*. As illustrated in Section S1.8.1 and Section S1.8.2, the mean pairwise graphlet correlation distance-11 (GCD-11) between "aquatic" webs constructed via Ecopath is marginally reduced/improved as compared to non-Ecopath "aquatic" webs (2.78 vs. 3.02, respectively). In contrast, the mean pairwise GCD-11 between food webs sourced from the same publication is much smaller and more similar, i.e., mean pairwise GCD-11: 1.51 (Table 2).

Of the non-Ecopath "aquatic" webs available from our dataset, 37 webs were sourced from publications that each provided only a single network, while 102 webs were sourced from 12 publications that each provided multiple networks (see Table S7 for list). Specifically, 3 webs were sourced from Closs and Lake (1994), 4 webs were sourced from Thompson and Townsend (2003), 2 webs were sourced from Alcorlo et al. (2001), 2 webs were sourced from Menge and Sutherland (1976), 2 webs were sourced from Tavares-Cromar and Williams (1996), 4 webs were sourced from Parker and Huryn (2006), 30 webs were sourced from Thompson and Townsend (2004), 2 webs were sourced from Cohen et al. (1993), 4 webs were sourced from Fryer (1959), 36 webs were sourced from Havens (1992), 9 were sourced from Layer et al. (2010), and 4 were sourced from O'Gorman et al. (2019).

Section S1.8.1 Structural similarity between "aquatic" food webs not constructed via Ecopath

Here, we evaluated the mean pairwise GCD-11 between all "aquatic" food webs not constructed using Ecopath. Since food webs sourced from the same publication were already known to be very highly structurally similar to each other (i.e., mean pairwise GCD-11 of 1.51 [Table 2]), we needed to remove publication effect from this Ecopath network analysis. To do so, we only included in our analyses, webs that each had unique publication sources. As a means of reducing the burden of sampling across all possible different and unique combinations of taking a single web from each of the 12 publications that provided multiple networks, we simply randomly chose 12 webs. We refer to a collated combination of 12 randomly chosen webs (each one from a unique publication source that provided multiple networks), and all 37 webs sourced from publications that each provided only a single network, as a single realization, which consisted of 49 webs. Across 200 realizations, we found the average mean pairwise GCD-11 between "aquatic" webs not constructed via Ecopath was 3.02.

Section S1.8.2 Structural similarity between "aquatic" food webs constructed via Ecopath

To test for the possible improvement in food web structural similarity when limiting one's analysis to only those that have been constructed using similar methodology, we also evaluated the mean pairwise GCD-11 between "aquatic" food webs constructed using Ecopath. Of the 28 "aquatic" food webs constructed using Ecopath (see Table S7 for list), 15 webs were sourced from publications that each produced only a single network, while 13 webs were sourced from 4 publications that each produced multiple networks. Specifically, 2 webs were sourced from An-

gelini et al. (2006), 3 webs were sourced from Angelini et al. (2013), 6 webs were sourced from Baeta et al. (2011), and 2 webs were sourced from Stewart and Sprules (2011).

Again, since food webs sourced from the same publication were very highly structurally similar to each other, we needed to remove this publication effect in order to effectively evaluate the structural similarity between the 28 “aquatic” webs constructed using Ecopath. To do so, we only included in our analyses webs that each had unique publication sources. Thus, we evaluated the mean pairwise GCD-11 between food webs sourced from publications that each produced only a single network, along with all 72 different unique combinations when including four chosen webs, one from each of the publications that produced multiple networks. We refer to a single unique combination of 4 webs each chosen from a different publication that produced multiple networks and the 15 webs sourced from publications that each produced a single network, as a single realization, which consisted of 19 webs. Across all 72 realizations, we found the average mean pairwise GCD-11 between “aquatic” food webs constructed via Ecopath was 2.78.

Section S1.9 Food web citations

Table S7: The list of 148 food webs used in this study. Webs are classified as belonging to type aquatic ("A"), aquatic and terrestrial ("A&T"), or terrestrial ("T").

#	Name	Type	Refined aquatic type	Publication grouping	Ecopath	Citation
1	WEB3_	A&T		One network per publication	NA	Woodwell (1967)
2	WEB4_	A&T		One network per publication	NA	Johnston (1956)
3	WEB6_	A&T		One network per publication	NA	MacGinitie (1935)
4	WEB12_	A	Marine	Menge & Sutherland (1976)	NA	Menge and Sutherland (1976)
5	WEB13_	A	Marine	Menge & Sutherland (1976)	NA	Menge and Sutherland (1976)
6	WEB17_	A	Marine	One network per publication	NA	Hiatt and Strasburg (1960)
7	WEB18_	A&T		One network per publication	NA	Niering (1963)
8	WEB22_	A&T		One network per publication	NA	Summerhayes and Elton (1923)
9	WEB28_	T		One network per publication	NA	Paviour-Smith (1956)
10	WEB29_	A	Marine	One network per publication	NA	Dunbar (1953)
11	WEB33_	A	Lake	Fryer (1959)	NA	Fryer (1959)
12	WEB204_	A	Lake	Fryer (1959)	NA	Fryer (1959)
13	WEB38_	A	Lake	Fryer (1959)	NA	Fryer (1959)
14	WEB39_	A	Lake	Fryer (1959)	NA	Fryer (1959)
15	WEB34_	A	Stream	One network per publication	NA	Erichsen Jones (1949)
16	WEB35_	A	Stream	One network per publication	NA	Minshall (1967)
17	WEB37_	A	Marine	One network per publication	NA	Clarke et al. (1967)
18	WEB40_	T		One network per publication	NA	Harrison (1962)
19	WEB42_	A	Marine	One network per publication	NA	Vinogradov and Shushkina (1978)
20	WEB43_	A	Marine	One network per publication	NA	Rosenthal et al. (1974)
21	WEB45_	A	Spring	One network per publication	NA	Tilly (1968)
22	WEB58_	A&T		One network per publication	NA	Smirnov (1961)
23	WEB59_	T		One network per publication	NA	Twomey (1945)
24	WEB63_	A	River	One network per publication	NA	Erichsen Jones (1950)
25	WEB67_	A&T		One network per publication	NA	Carlson (1968)
26	WEB72_	A	Lake	One network per publication	NA	Baril (1983)
27	WEB84_	A&T		One network per publication	NA	Wilbur (1972)
28	WEB87_	A	Marine	One network per publication	NA	Bradstreet and Cross (1982)
29	WEB88_	A	River	One network per publication	NA	Kuusela (1979)

Table continued . . .

. . . Continuation of Table S7.

#	Name	Type	Refined aquatic type	Publication grouping	Ecopath	Citation
30	WEB89_	A	River	One network per publication	NA	Hartley (1948)
31	WEB98_	T		One network per publication	NA	Holm and Scholtz (1980)
32	WEB104_	A	Marine	One network per publication	NA	Menge et al. (1986)
33	WEB105_	A	Marine	One network per publication	NA	Edwards et al. (1982)
34	WEB107_	A	Marine	One network per publication	NA	Peterson (1979)
35	WEB108_	A	Marine	One network per publication	NA	Hewatt (1937)
36	WEB117_	A	Lake	One network per publication	NA	Zaret and Paine (1973)
37	WEB121_	A	Marine	One network per publication	NA	van Es (1977)
38	WEB123_	T		One network per publication	NA	Harris and Paur (1972)
39	WEB131_	A&T		Beaver (1985)	NA	Beaver (1985)
40	WEB132_	A&T		Beaver (1985)	NA	Beaver (1985)
41	WEB134_	A&T		Beaver (1985)	NA	Beaver (1985)
42	WEB151_	T		One network per publication	NA	Richards (1926)
43	WEB152_	T		One network per publication	NA	Whittaker (1984)
44	WEB154_	T		One network per publication	NA	Mayse and Price (1978)
45	WEB155_	T		One network per publication	NA	Askew (1975)
46	WEB199_	T		One network per publication	NA	Valiela (1969)
47	WEB200_	T		Valiela (1974)	NA	Valiela (1974)
48	WEB201_	T		Valiela (1974)	NA	Valiela (1974)
49	WEB205_	A	Stream	One network per publication	NA	Hildrew et al. (1985)
50	WEB207_	A	Stream	One network per publication	NA	Koslucher and Minshall (1973)
51	WEB208_	A	Stream	One network per publication	NA	Minckley (1963)
52	WEB210_	A	Stream	One network per publication	NA	Percival and Whitehead (1929)
53	WEB211_	A	Stream	One network per publication	NA	Ricker (1934)
54	WEB213_	A	Stream	One network per publication	NA	Badcock (1949)
55	WEB214_	A	Stream	Thompson & Townsend (2003)	NA	Thompson and Townsend (2003)
56	WEB215_	A	Stream	Thompson & Townsend (2003)	NA	Thompson and Townsend (2003)
57	WEB216_	A	Stream	Thompson & Townsend (2003)	NA	Thompson and Townsend (2003)
58	WEB217_	A	Stream	Thompson & Townsend (2003)	NA	Thompson and Townsend (2003)
59	WEB218_	A	Stream	Thompson & Townsend (2004)	NA	Thompson and Townsend (2004)
60	WEB219_	A	Stream	Thompson & Townsend (2004)	NA	Thompson and Townsend (2004)
61	WEB220_	A	Stream	Thompson & Townsend (2004)	NA	Thompson and Townsend (2004)
62	WEB221_	A	Stream	Thompson & Townsend (2004)	NA	Thompson and Townsend (2004)
63	WEB222_	A	Stream	Thompson & Townsend (2004)	NA	Thompson and Townsend (2004)

Table continued . . .

...Continuation of Table S7.

#	Name	Type	Refined aquatic type	Publication grouping	Ecopath	Citation
64	WEB223_	A	Stream	Thompson & Townsend (2004)	NA	Thompson and Townsend (2004)
65	WEB224_	A	Stream	Thompson & Townsend (2004)	NA	Thompson and Townsend (2004)
66	WEB225_	A	Stream	Thompson & Townsend (2004)	NA	Thompson and Townsend (2004)
67	WEB226_	A	Stream	Thompson & Townsend (2004)	NA	Thompson and Townsend (2004)
68	WEB227_	A	Stream	Thompson & Townsend (2004)	NA	Thompson and Townsend (2004)
69	WEB228_	A	Stream	Thompson & Townsend (2004)	NA	Thompson and Townsend (2004)
70	WEB229_	A	Stream	Thompson & Townsend (2004)	NA	Thompson and Townsend (2004)
71	WEB230_	A	Stream	Thompson & Townsend (2004)	NA	Thompson and Townsend (2004)
72	WEB231_	A	Stream	Thompson & Townsend (2004)	NA	Thompson and Townsend (2004)
73	WEB232_	A	Stream	Thompson & Townsend (2004)	NA	Thompson and Townsend (2004)
74	WEB233_	A	Stream	Thompson & Townsend (2004)	NA	Thompson and Townsend (2004)
75	WEB234_	A	Stream	Thompson & Townsend (2004)	NA	Thompson and Townsend (2004)
76	WEB235_	A	Stream	Thompson & Townsend (2004)	NA	Thompson and Townsend (2004)
77	WEB236_	A	Stream	Thompson & Townsend (2004)	NA	Thompson and Townsend (2004)
78	WEB237_	A	Stream	Thompson & Townsend (2004)	NA	Thompson and Townsend (2004)
79	WEB238_	A	Stream	Thompson & Townsend (2004)	NA	Thompson and Townsend (2004)
80	WEB239_	A	Stream	Thompson & Townsend (2004)	NA	Thompson and Townsend (2004)
81	WEB240_	A	Stream	Thompson & Townsend (2004)	NA	Thompson and Townsend (2004)
82	WEB241_	A	Stream	Thompson & Townsend (2004)	NA	Thompson and Townsend (2004)
83	WEB242_	A	Stream	Thompson & Townsend (2004)	NA	Thompson and Townsend (2004)
84	WEB243_	A	Stream	Thompson & Townsend (2004)	NA	Thompson and Townsend (2004)
85	WEB244_	A	Stream	Thompson & Townsend (2004)	NA	Thompson and Townsend (2004)
86	WEB245_	A	Stream	Thompson & Townsend (2004)	NA	Thompson and Townsend (2004)
87	WEB246_	A	Stream	Thompson & Townsend (2004)	NA	Thompson and Townsend (2004)
88	WEB247_	A	Stream	Thompson & Townsend (2004)	NA	Thompson and Townsend (2004)
89	WEB248_	A&T		One network per publication	NA	Thompson et al. (2005)
90	WEB249_	A	River	One network per publication	Yes	Angelini and Agostinho (2005)
91	WEB250_	A	River	Angelini et al. (2006)	Yes	Angelini et al. (2006)
92	WEB251_	A	River	Angelini et al. (2006)	Yes	Angelini et al. (2006)
93	WEB252_	A	River	One network per publication	Yes	Angelini et al. (2010)
94	WEB253_	A	Marine	One network per publication	Yes	Angelini and Vaz-Velho (2011)
95	WEB254_	A	Lake	Angelini et al. (2013)	Yes	Angelini et al. (2013)
96	WEB255_	A	Lake	Angelini et al. (2013)	Yes	Angelini et al. (2013)
97	WEB256_	A	Lake	Angelini et al. (2013)	Yes	Angelini et al. (2013)

Table continued ...

. . . Continuation of Table S7.

#	Name	Type	Refined aquatic type	Publication grouping	Ecopath	Citation
98	WEB257_	A	Marine	Baeta et al. (2011)	Yes	Baeta et al. (2011)
99	WEB258_	A	Marine	Baeta et al. (2011)	Yes	Baeta et al. (2011)
100	WEB259_	A	Marine	Baeta et al. (2011)	Yes	Baeta et al. (2011)
101	WEB260_	A	Marine	Baeta et al. (2011)	Yes	Baeta et al. (2011)
102	WEB261_	A	Marine	Baeta et al. (2011)	Yes	Baeta et al. (2011)
103	WEB262_	A	Marine	Baeta et al. (2011)	Yes	Baeta et al. (2011)
104	WEB263_	A&T		One network per publication	NA	Schneider (1997)
105	WEB264_	A	Stream	One network per publication	NA	Stagliano and Whiles (2002)
106	WEB265_	A	Marine	One network per publication	Yes	Lin et al. (2006)
107	WEB266_	A	Marine	One network per publication	Yes	Cornejo-Donoso and Antezana (2008)
108	WEB267_	A	Marine	One network per publication	Yes	Zetina-Rejón et al. (2003)
109	WEB268_	A	Marine	One network per publication	Yes	Cruz-Escalona et al. (2007)
110	WEB269_	A	Lake	One network per publication	Yes	Liu et al. (2007)
111	WEB270_	A	Marine	One network per publication	NA	Filgueira and Castro (2011)
112	WEB271_	A	Lake	One network per publication	NA	Amundsen et al. (2013)
113	WEB273_	A	Stream	Parker & Huryn (2006)	NA	Parker and Huryn (2006)
114	WEB274_	A	Stream	Parker & Huryn (2006)	NA	Parker and Huryn (2006)
115	WEB275_	A	Stream	Parker & Huryn (2006)	NA	Parker and Huryn (2006)
116	WEB276_	A	Stream	Parker & Huryn (2006)	NA	Parker and Huryn (2006)
117	WEB278_	A	Lake	Stewart & Sprules (2011)	Yes	Stewart and Sprules (2011)
118	WEB279_	A	Lake	Stewart & Sprules (2011)	Yes	Stewart and Sprules (2011)
119	WEB281_	A	Stream	Tavares-Cromar & Williams (1996)	NA	Tavares-Cromar and Williams (1996)
120	WEB285_	A	Stream	Tavares-Cromar & Williams (1996)	NA	Tavares-Cromar and Williams (1996)
121	WEB288_	A	Marine	One network per publication	Yes	Christian and Luczkovich (1999)
122	WEB289_	A	Lake	One network per publication	Yes	Fetahi et al. (2011)
123	WEB295_	A&T		One network per publication	NA	Preston et al. (2012)
124	WEB296_	A&T		One network per publication	NA	Ratsirarson and Silander (1996)
125	WEB306_	A	Stream	Closs & Lake (1994)	NA	Closs and Lake (1994)
126	WEB307_	A	Stream	Closs & Lake (1994)	NA	Closs and Lake (1994)
127	WEB308_	A	Stream	Closs & Lake (1994)	NA	Closs and Lake (1994)
128	WEB309_	A	Marine	One network per publication	NA	Gontikaki et al. (2011)
129	WEB310_	A	River	One network per publication	Yes	Khan and Panikkar (2009)
130	WEB311_	T		One network per publication	NA	Memmott et al. (2000)
131	WEB334_	A	Lake	Alcorlo et al. (2001)	NA	Alcorlo et al. (2001)

Table continued . . .

. . . Continuation of Table S7.

#	Name	Type	Refined aquatic type	Publication grouping	Ecopath	Citation
132	WEB335_	A	Lake	Alcorlo et al. (2001)	NA	Alcorlo et al. (2001)
133	WEB338_	A	Marine	One network per publication	Yes	Torres et al. (2013)
134	WEB340_	A	Marine	One network per publication	NA	Smith et al. (2007)
135	WEB343_	A&T		One network per publication	NA	Kitching (1987)
136	WEB344_	T		One network per publication	NA	Hodkinson and Coulson (2004)
137	WEB345_	A	Lake	One network per publication	NA	Boit et al. (2012)
138	WEB347_	A	Stream	One network per publication	NA	Motta and Uieda (2005)
139	WEB350_	A	Marine	One network per publication	NA	Douglass et al. (2011)
140	WEB351_	A&T		One network per publication	NA	Warren (1989)
141	WEB353_	A	Stream	One network per publication	Yes	Poepperl (2003)
142	WEB354_	T		One network per publication	NA	Goldwasser and Roughgarden (1993)
143	WEB355_	A	Lake	Cohen et al. (2003)	NA	Cohen et al. (2003)
144	WEB356_	A	Lake	Cohen et al. (2003)	NA	Cohen et al. (2003)
145	WEB357_	A	Marine	One network per publication	NA	Yodzis (1998)
146	WEB358_	T		One network per publication	NA	Schröter et al. (2003)
147	carpinteria_	A&T		One network per publication	NA	Lafferty et al. (2006)
148	FW_008_	A	Marine	One network per publication	Yes	Bascompte et al. (2005)
149	mown_Clmown1_	T		Cattin Blandenier (2004)	NA	Cattin Blandenier (2004)
150	mown_Clmown2_	T		Cattin Blandenier (2004)	NA	Cattin Blandenier (2004)
151	mown_Scmown1_	T		Cattin Blandenier (2004)	NA	Cattin Blandenier (2004)
152	mown_Scmown2_	T		Cattin Blandenier (2004)	NA	Cattin Blandenier (2004)
153	not_mown_ClControl1_	T		Cattin Blandenier (2004)	NA	Cattin Blandenier (2004)
154	not_mown_ClControl2_	T		Cattin Blandenier (2004)	NA	Cattin Blandenier (2004)
155	not_mown_ScControl1_	T		Cattin Blandenier (2004)	NA	Cattin Blandenier (2004)
156	not_mown_ScControl2_	T		Cattin Blandenier (2004)	NA	Cattin Blandenier (2004)
157	AEW01_	T		Digel et al. (2014)	NA	Digel et al. (2014)
158	AEW02_	T		Digel et al. (2014)	NA	Digel et al. (2014)
159	AEW03_	T		Digel et al. (2014)	NA	Digel et al. (2014)
160	AEW04_	T		Digel et al. (2014)	NA	Digel et al. (2014)
161	AEW05_	T		Digel et al. (2014)	NA	Digel et al. (2014)
162	AEW06_	T		Digel et al. (2014)	NA	Digel et al. (2014)
163	AEW07_	T		Digel et al. (2014)	NA	Digel et al. (2014)
164	AEW08_	T		Digel et al. (2014)	NA	Digel et al. (2014)
165	AEW09_	T		Digel et al. (2014)	NA	Digel et al. (2014)

Table continued . . .

. . . Continuation of Table S7.

#	Name	Type	Refined aquatic type	Publication grouping	Ecopath	Citation
166	AEW11_	T		Digel et al. (2014)	NA	Digel et al. (2014)
167	AEW17_	T		Digel et al. (2014)	NA	Digel et al. (2014)
168	AEW18_	T		Digel et al. (2014)	NA	Digel et al. (2014)
169	AEW25_	T		Digel et al. (2014)	NA	Digel et al. (2014)
170	AEW27_	T		Digel et al. (2014)	NA	Digel et al. (2014)
171	AEW30_	T		Digel et al. (2014)	NA	Digel et al. (2014)
172	AEW49_	T		Digel et al. (2014)	NA	Digel et al. (2014)
173	HEW01_	T		Digel et al. (2014)	NA	Digel et al. (2014)
174	HEW02_	T		Digel et al. (2014)	NA	Digel et al. (2014)
175	HEW03_	T		Digel et al. (2014)	NA	Digel et al. (2014)
176	HEW04_	T		Digel et al. (2014)	NA	Digel et al. (2014)
177	HEW05_	T		Digel et al. (2014)	NA	Digel et al. (2014)
178	HEW06_	T		Digel et al. (2014)	NA	Digel et al. (2014)
179	HEW10_	T		Digel et al. (2014)	NA	Digel et al. (2014)
180	HEW11_	T		Digel et al. (2014)	NA	Digel et al. (2014)
181	HEW12_	T		Digel et al. (2014)	NA	Digel et al. (2014)
182	HEW13_	T		Digel et al. (2014)	NA	Digel et al. (2014)
183	HEW16_	T		Digel et al. (2014)	NA	Digel et al. (2014)
184	HEW17_	T		Digel et al. (2014)	NA	Digel et al. (2014)
185	HEW21_	T		Digel et al. (2014)	NA	Digel et al. (2014)
186	HEW22_	T		Digel et al. (2014)	NA	Digel et al. (2014)
187	HEW36_	T		Digel et al. (2014)	NA	Digel et al. (2014)
188	HEW47_	T		Digel et al. (2014)	NA	Digel et al. (2014)
189	SEW01_	T		Digel et al. (2014)	NA	Digel et al. (2014)
190	SEW02_	T		Digel et al. (2014)	NA	Digel et al. (2014)
191	SEW03_	T		Digel et al. (2014)	NA	Digel et al. (2014)
192	SEW04_	T		Digel et al. (2014)	NA	Digel et al. (2014)
193	SEW05_	T		Digel et al. (2014)	NA	Digel et al. (2014)
194	SEW06_	T		Digel et al. (2014)	NA	Digel et al. (2014)
195	SEW07_	T		Digel et al. (2014)	NA	Digel et al. (2014)
196	SEW08_	T		Digel et al. (2014)	NA	Digel et al. (2014)
197	SEW09_	T		Digel et al. (2014)	NA	Digel et al. (2014)
198	SEW18_	T		Digel et al. (2014)	NA	Digel et al. (2014)
199	SEW35_	T		Digel et al. (2014)	NA	Digel et al. (2014)

Table continued . . .

. . . Continuation of Table S7.

#	Name	Type	Refined aquatic type	Publication grouping	Ecopath	Citation
200	SEW36_	T		Digel et al. (2014)	NA	Digel et al. (2014)
201	SEW37_	T		Digel et al. (2014)	NA	Digel et al. (2014)
202	SEW41_	T		Digel et al. (2014)	NA	Digel et al. (2014)
203	SEW43_	T		Digel et al. (2014)	NA	Digel et al. (2014)
204	SEW48_	T		Digel et al. (2014)	NA	Digel et al. (2014)
205	Alford_Lake_	A	Lake	Havens (1992)	NA	Havens (1992)
206	Balsam_Lake_	A	Lake	Havens (1992)	NA	Havens (1992)
207	Beaver_Lake_	A	Lake	Havens (1992)	NA	Havens (1992)
208	Big_Hope_Lake_	A	Lake	Havens (1992)	NA	Havens (1992)
209	Brandy_Lake_	A	Lake	Havens (1992)	NA	Havens (1992)
210	Bridge_Brook_Lake_	A	Lake	Havens (1992)	NA	Havens (1992)
211	Burntbridge_Lake_	A	Lake	Havens (1992)	NA	Havens (1992)
212	Cascade_Lake_	A	Lake	Havens (1992)	NA	Havens (1992)
213	Chub_Lake_	A	Lake	Havens (1992)	NA	Havens (1992)
214	Connera_Lake_	A	Lake	Havens (1992)	NA	Havens (1992)
215	Constable_Lake_	A	Lake	Havens (1992)	NA	Havens (1992)
216	Emerald_Lake_	A	Lake	Havens (1992)	NA	Havens (1992)
217	Falls_Lake_	A	Lake	Havens (1992)	NA	Havens (1992)
218	Fawn_Lake_	A	Lake	Havens (1992)	NA	Havens (1992)
219	Federation_Lake_	A	Lake	Havens (1992)	NA	Havens (1992)
220	Goose_Lake_	A	Lake	Havens (1992)	NA	Havens (1992)
221	Grass_Lake_	A	Lake	Havens (1992)	NA	Havens (1992)
222	Gull_Lake_	A	Lake	Havens (1992)	NA	Havens (1992)
223	Hoel_Lake_	A	Lake	Havens (1992)	NA	Havens (1992)
224	Horseshoe_Lake_	A	Lake	Havens (1992)	NA	Havens (1992)
225	Little_Rainbow_Lake_	A	Lake	Havens (1992)	NA	Havens (1992)
226	Long_Lake_	A	Lake	Havens (1992)	NA	Havens (1992)
227	Loon_Lake_	A	Lake	Havens (1992)	NA	Havens (1992)
228	Lost_Lake_East_	A	Lake	Havens (1992)	NA	Havens (1992)
229	Lost_Lake_	A	Lake	Havens (1992)	NA	Havens (1992)
230	Lower_Sister_Lake_	A	Lake	Havens (1992)	NA	Havens (1992)
231	Oswego_Lake_	A	Lake	Havens (1992)	NA	Havens (1992)
232	Rat_Lake_	A	Lake	Havens (1992)	NA	Havens (1992)
233	Razorback_Lake_	A	Lake	Havens (1992)	NA	Havens (1992)

Table continued . . .

. . . Continuation of Table S7.

#	Name	Type	Refined aquatic type	Publication grouping	Ecopath	Citation
234	Russian_Lake_	A	Lake	Havens (1992)	NA	Havens (1992)
235	Safford_Lake_	A	Lake	Havens (1992)	NA	Havens (1992)
236	Sand_Lake_	A	Lake	Havens (1992)	NA	Havens (1992)
237	Squaw_Lake_	A	Lake	Havens (1992)	NA	Havens (1992)
238	Stink_Lake_	A	Lake	Havens (1992)	NA	Havens (1992)
239	Twelfth_Tee_Lake_	A	Lake	Havens (1992)	NA	Havens (1992)
240	Whipple_Lake_	A	Lake	Havens (1992)	NA	Havens (1992)
241	Buck_Pond_	A&T		Havens (1992)	NA	Havens (1992)
242	Chub_Pond_	A&T		Havens (1992)	NA	Havens (1992)
243	Helldiver_Pond_	A&T		Havens (1992)	NA	Havens (1992)
244	High_Pond_	A&T		Havens (1992)	NA	Havens (1992)
245	Afon_Hafren_2005_	A	Stream	Layer et al. (2010)	NA	Layer et al. (2010)
246	Allt_a_Mharcaidh_	A	Stream	Layer et al. (2010)	NA	Layer et al. (2010)
247	Broadstone_Stream_	A	Stream	Layer et al. (2010)	NA	Layer et al. (2010)
248	Dargall_Lane_	A	Stream	Layer et al. (2010)	NA	Layer et al. (2010)
249	Duddon_Pike_Beck_	A	Stream	Layer et al. (2010)	NA	Layer et al. (2010)
250	Hardknott_Gill_	A	Stream	Layer et al. (2010)	NA	Layer et al. (2010)
251	Mill_Stream_	A	Stream	Layer et al. (2010)	NA	Layer et al. (2010)
252	Mosendale_Beck_	A	Stream	Layer et al. (2010)	NA	Layer et al. (2010)
253	Old_Lodge_	A	Stream	Layer et al. (2010)	NA	Layer et al. (2010)
254	Alert_	T		Legagneux et al. (2014)	NA	Legagneux et al. (2014)
255	Bylot_	T		Legagneux et al. (2014)	NA	Legagneux et al. (2014)
256	Herschel_	T		Legagneux et al. (2014)	NA	Legagneux et al. (2014)
257	Nenetsky_	T		Legagneux et al. (2014)	NA	Legagneux et al. (2014)
258	Yamal_	T		Legagneux et al. (2014)	NA	Legagneux et al. (2014)
259	Zackenberg_	T		Legagneux et al. (2014)	NA	Legagneux et al. (2014)
260	Iceland_Stream_IS7 _April_2009_	A	Stream	O'Gorman et al. (2019)	NA	O'Gorman et al. (2019)
261	Iceland_Stream_IS7 _August_2008_	A	Stream	O'Gorman et al. (2019)	NA	O'Gorman et al. (2019)
262	Iceland_Stream_IS8 _April_2009_	A	Stream	O'Gorman et al. (2019)	NA	O'Gorman et al. (2019)
263	Iceland_Stream_IS8 _August_2008_	A	Stream	O'Gorman et al. (2019)	NA	O'Gorman et al. (2019)

Table continued . . .

. . . Continuation of Table S7.

#	Name	Type	Refined aquatic type	Publication grouping	Ecopath	Citation
264	Lake_Malawi_	A	Lake	One network per publication	Yes	Nsiku (1999)
265	Ythan_Estuary_	A	Marine	One network per publication	NA	Cohen et al. (2009)
266	Kongsfjorden_	A	Marine	One network per publication	NA	Eklöf et al. (2013)
267	Weddell_Sea_	A	Marine	One network per publication	NA	Jacob et al. (2011)
268	Skipwith_Pond_	A&T		One network per publication	NA	Warren (1990)
269	Gearagh_	T		One network per publication	NA	McLaughlin et al. (2010)
270	FloridaIslandE1_	A&T		Piechnik et al. (2008)	NA	Piechnik et al. (2008)
271	FloridaIslandE2_	A&T		Piechnik et al. (2008)	NA	Piechnik et al. (2008)
272	FloridaIslandE3_	A&T		Piechnik et al. (2008)	NA	Piechnik et al. (2008)
273	FloridaIslandE7_	A&T		Piechnik et al. (2008)	NA	Piechnik et al. (2008)
274	FloridaIslandE9_	A&T		Piechnik et al. (2008)	NA	Piechnik et al. (2008)

Food web citations

Alcorlo, P., A. Baltanás, and C. Montes. 2001. Food-web structure in two shallow salt lakes in Los Monegros (NE Spain): Energetic vs dynamic constraints. In J. M. Melack, R. Jellison, and D. B. Herbst, editors, Saline Lakes: Publications from the 7th International Conference on Salt Lakes, held in Death Valley National Park, California, U.S.A., September 1999, pages 307–316. Springer Netherlands, Dordrecht.

Amundsen, P.-A., K. D. Lafferty, R. Knudsen, R. Primicerio, R. Kristoffersen, A. Klemetsen, and A. M. Kuris. 2013. New parasites and predators follow the introduction of two fish species to a subarctic lake: Implications for food-web structure and functioning. Oecologia, **171**:993–1002.

Angelini, R. and A. A. Agostinho. 2005. Food web model of the Upper Paraná river floodplain: Description and aggregation effects. Ecological Modelling, **181**:109–121.

Angelini, R., A. A. Agostinho, and L. C. Gomes. 2006. Modeling energy flow in a large neotropical reservoir: A tool do evaluate fishing and stability. Neotropical Ichthyology, **4**:253–260.

Angelini, R., G. R. Aloisio, and A. R. Carvalho. 2010. Mixed food web control and stability in a Cerrado river (Brazil). Pan-American Journal of Aquatic Sciences, **5**:421–431.

Angelini, R., R. J. de Morais, A. C. Catella, E. K. Resende, and S. Libralato. 2013. Aquatic food webs of the oxbow lakes in the Pantanal: A new site for fisheries guaranteed by alternated control? Ecological Modelling, **253**:82–96.

Angelini, R. and F. Vaz-Velho. 2011. Ecosystem structure and trophic analysis of Angolan fishery landings. Scientia Marina, **75**:309–319.

Askew, R. R. 1975. The organisation of Chalcid-dominated parasitoid communities centred upon endophytic hosts. In P. W. Price, editor, Evolutionary Strategies of Parasitic Insects and Mites, pages 130–153. Springer US, Boston, MA.

Badcock, R. M. 1949. Studies in stream life in tributaries of the Welsh Dee. Journal of Animal Ecology, **18**:193–208.

Baeta, A., N. Niquil, J. C. Marques, and J. Patrício. 2011. Modelling the effects of eutrophication, mitigation measures and an extreme flood event on estuarine benthic food webs. Ecological Modelling, **222**:1209–1221.

Baril, A. 1983. Effect of the water mite Piona constricta on planktonic community structure. Thesis, University of Ottawa.

Bascompte, J., C. J. Melián, and E. Sala. 2005. Interaction strength combinations and the overfishing of a marine food web. Proceedings of the National Academy of Sciences, **102**:5443–5447.

Beaver, R. A. 1985. Geographical variation in food web structure in Nepenthes pitcher plants. Ecological Entomology, **10**:241–248.

Boit, A., N. D. Martinez, R. J. Williams, and U. Gaedke. 2012. Mechanistic theory and modelling of complex food-web dynamics in Lake Constance. Ecology Letters, **15**:594–602.

Bradstreet, M. S. W. and W. E. Cross. 1982. Trophic relationships at high Arctic ice edges. Arctic, **35**:1–12.

Carlson, C. A. 1968. Summer bottom fauna of the Mississippi River, above dam 19, Keokuk, Iowa. Ecology, **49**:162–169.

Cattin Blandenier, M.-F. 2004. Food web ecology: Models and application to conservation. Thesis, Université de Neuchâtel.

Christian, R. R. and J. J. Luczkovich. 1999. Organizing and understanding a winter's seagrass foodweb network through effective trophic levels. Ecological Modelling, **117**:99–124.

Clarke, T. A., A. O. Fleehsig, and R. W. Grigg. 1967. Ecological studies during Project Sealab II. Science, **157**:1381–1389.

Closs, G. P. and P. S. Lake. 1994. Spatial and temporal variation in the structure of an intermittent-stream food web. Ecological Monographs, **64**:1–21.

Cohen, J. E., T. Jonsson, and S. R. Carpenter. 2003. Ecological community description using the food web, species abundance, and body size. Proceedings of the National Academy of Sciences, **100**:1781–1786.

Cohen, J. E., D. N. Schittler, D. G. Raffaelli, and D. C. Reuman. 2009. Food webs are more than the sum of their tritrophic parts. Proceedings of the National Academy of Sciences, **106**:22335–22340.

Cornejo-Donoso, J. and T. Antezana. 2008. Preliminary trophic model of the Antarctic Peninsula Ecosystem (sub-area CCAMLR 48.1). Ecological Modelling, **218**:1–17.

Cruz-Escalona, V., F. Arreguín-Sánchez, and M. Zetina-Rejón. 2007. Analysis of the ecosystem structure of Laguna Alvarado, western Gulf of Mexico, by means of a mass balance model. Estuarine, Coastal and Shelf Science, **72**:155–167.

Digel, C., A. Curtsdotter, J. Riede, B. Klarner, and U. Brose. 2014. Unravelling the complex structure of forest soil food webs: higher omnivory and more trophic levels. Oikos, **123**:1157–1172.

Douglass, J. G., J. E. Duffy, and E. A. Canuel. 2011. Food web structure in a Chesapeake Bay eelgrass bed as determined through gut contents and ^{13}C and ^{15}N isotope analysis. Estuaries and Coasts, **34**:701–711.

Dunbar, M. J. 1953. Arctic and subarctic marine ecology: Immediate problems. Arctic, **6**:75–90.

Edwards, D. C., D. O. Conover, and F. Sutter III. 1982. Mobile predators and the structure of marine intertidal communities. Ecology, **63**:1175–1180.

Eklöf, A., U. Jacob, J. Kopp, J. Bosch, R. Castro-Urgal, N. P. Chacoff, B. Dalsgaard, C. de Sassi, M. Galetti, P. R. Guimarães, S. B. Lomáscolo, A. M. Martìn González, M. A. Pizo, R. Rader, A. Rodrigo, J. M. Tylianakis, D. P. Vázquez, and S. Allesina. 2013. The dimensionality of ecological networks. Ecology Letters, **16**:577–583.

Erichsen Jones, J. R. 1949. A further ecological study of calcareous streams in the 'Black Mountain' district of South Wales. Journal of Animal Ecology, **18**:142–159.

Erichsen Jones, J. R. 1950. A further ecological study of the River Rheidol: The food of the common insects of the main-stream. Journal of Animal Ecology, **19**:159–174.

Fetahi, T., M. Schagerl, S. Mengistou, and S. Libralato. 2011. Food web structure and trophic interactions of the tropical highland lake Hayq, Ethiopia. Ecological Modelling, **222**:804–813.

Filgueira, R. and B. G. Castro. 2011. Study of the trophic web of San Simón Bay (Ría de Vigo) by using stable isotopes. Continental Shelf Research, **31**:476–487.

Fryer, G. 1959. The trophic interrelationships and ecology of some littoral communities of Lake Nyasa with especial reference to the fishes, and a discussion of the evolution of a group of rock-frequenting Cichlidae. Proceedings of the Zoological Society of London, **132**:153–281.

Goldwasser, L. and J. Roughgarden. 1993. Construction and analysis of a large Caribbean food web. Ecology, **74**:1216–1233.

Gontikaki, E., D. van Oevelen, K. Soetaert, and U. Witte. 2011. Food web flows through a subarctic deep-sea benthic community. Progress in Oceanography, **91**:245–259.

Harris, L. D. and L. F. Paur. 1972. A quantitative food web analysis of a shortgrass community. Technical report, No. 154, Grassland Biome. (U.S. International Biological Program).

Harrison, J. L. 1962. The distribution of feeding habits among animals in a tropical rain forest. Journal of Animal Ecology, **31**:53–63.

Hartley, P. H. T. 1948. Food and feeding relationships in a community of fresh-water fishes. Journal of Animal Ecology, **17**:1–14.

Havens, K. 1992. Scale and structure in natural food webs. Science, **257**:1107–1109.

Hewatt, W. G. 1937. Ecological studies on selected marine intertidal communities of Monterey Bay, California. The American Midland Naturalist, **18**:161–206.

Hiatt, R. W. and D. W. Strasburg. 1960. Ecological relationships of the fish fauna on coral reefs of the Marshall Islands. Ecological Monographs, **30**:65–127.

Hildrew, A. G., C. R. Townsend, and A. Hasham. 1985. The predatory Chironomidae of an iron-rich stream: Feeding ecology and food web structure. Ecological Entomology, **10**:403–413.

Hodkinson, I. D. and S. J. Coulson. 2004. Are high arctic terrestrial food chains really that simple? – The Bear Island food web revisited. Oikos, **106**:427–431.

Holm, E. and C. H. Scholtz. 1980. Structure and pattern of the Namib Desert dune ecosystem at Gobabeb. Madoqua, **1980**:3–39.

Jacob, U., A. Thierry, U. Brose, W. E. Arntz, S. Berg, T. Brey, I. Fetzer, T. Jonsson, K. Mintenbeck, C. Möllmann, O. L. Petchey, J. O. Riede, and J. A. Dunne. 2011. The role of body size in complex food webs: A cold case. In A. Belgrano, editor, The Role of Body Size in Multispecies Systems, volume 45 of *Advances in Ecological Research*, pages 181–223. Academic Press.

Johnston, R. F. 1956. Predation by short-eared owls on a salicornia salt marsh. The Wilson Bulletin, **68**:91–102.

Khan, M. F. and P. Panikkar. 2009. Assessment of impacts of invasive fishes on the food web structure and ecosystem properties of a tropical reservoir in India. Ecological Modelling, **220**:2281–2290.

Kitching, R. L. 1987. Spatial and temporal variation in food webs in water-filled treeholes. Oikos, **48**:280–288.

Koslucher, D. G. and G. W. Minshall. 1973. Food habits of some benthic invertebrates in a northern cool-desert stream (Deep Creek, Curlew Valley, Idaho-Utah). Transactions of the American Microscopical Society, **92**:441–452.

Kuusela, K. 1979. Early summer ecology and community structure of the macrozoobenthos on stones in the Jäväjänkoski Rapids on the River Lestijoki, Finland. Thesis, University of Oulu.

Lafferty, K. D., R. F. Hechinger, J. C. Shaw, K. L. Whitney, and A. M. Kuris. 2006. Food webs and parasites in a salt marsh ecosystem. In S. Collinge and C. Ray, editors, Disease ecology: Community structure and pathogen dynamics, pages 119—134. Oxford University Press, Oxford, UK.

Layer, K., J. O. Riede, A. G. Hildrew, and G. Woodward. 2010. Chapter 5 - food web structure and stability in 20 streams across a wide ph gradient. In G. Woodward, editor, Ecological Networks, volume 42 of *Advances in Ecological Research*, pages 265–299. Academic Press.

Legagneux, P., G. Gauthier, N. Lecomte, N. M. Schmidt, D. Reid, M.-C. Cadieux, D. Berteaux, J. Bêty, C. J. Krebs, R. A. Ims, N. G. Yoccoz, R. I. G. Morrison, S. J. , M. Loreau, and D. Gravel. 2014. Arctic ecosystem structure and functioning shaped by climate and herbivore body size. Nature Climate Change, **4**:379–383.

Lin, H.-J., X.-X. Dai, K.-T. Shao, H.-M. Su, W.-T. Lo, H.-L. Hsieh, L.-S. Fang, and J.-J. Hung. 2006. Trophic structure and functioning in a eutrophic and poorly flushed lagoon in southwestern Taiwan. Marine Environmental Research, **62**:61–82.

Liu, Q.-G., Y. Chen, J.-L. Li, and L.-Q. Chen. 2007. The food web structure and ecosystem properties of a filter-feeding carps dominated deep reservoir ecosystem. Ecological Modelling, **203**:279–289.

MacGinitie, G. E. 1935. Ecological aspects of a California marine estuary. The American Midland Naturalist, **16**:629–765.

Mayse, M. A. and P. W. Price. 1978. Seasonal development of soybean arthropod communities in east central Illinois. Agro-Ecosystems, **4**:387–405.

McLaughlin, O. B., T. Jonsson, and M. C. Emmerson. 2010. Chapter 4 - temporal variability in predator–prey relationships of a forest floor food web. In G. Woodward, editor, Ecological Networks, volume 42 of *Advances in Ecological Research*, pages 171–264. Academic Press.

Memmott, J., N. D. Martinez, and J. E. Cohen. 2000. Predators, parasitoids and pathogens: Species richness, trophic generality and body sizes in a natural food web. Journal of Animal Ecology, **69**:1–15.

Menge, B. A., J. Lubchenco, S. D. Gaines, and L. R. Ashkenas. 1986. A test of the Menge-Sutherland model of community organization in a tropical rocky intertidal food web. Oecologia, **71**:75–89.

Menge, B. A. and J. P. Sutherland. 1976. Species diversity gradients: Synthesis of the roles of predation, competition, and temporal heterogeneity. The American Naturalist, **110**:351–369.

Minckley, W. L. 1963. The ecology of a spring stream: Doe Run, Meade County, Kentucky. Wildlife Monographs, pages 3–124.

Minshall, G. W. 1967. Role of allochthonous detritus in the trophic structure of a woodland springbrook community. Ecology, **48**:139–149.

Motta, R. L. and V. S. Uieda. 2005. Food web structure in a tropical stream ecosystem. Austral Ecology, **30**:58–73.

Niering, W. A. 1963. Terrestrial ecology of Kapingamarangi Atoll, Caroline Islands. Ecological Monographs, **33**:131–160.

Nsiku, E. 1999. Changes in the fisheries of Lake Malawi, 1976—1996: Ecosystem-based analysis. Thesis, The University of British Columbia.

O'Gorman, E. J., O. L. Petchey, K. J. Faulkner, B. Gallo, T. A. C. Gordon, J. Neto-Cerejeira, J. S. Ólafsson, D. E. Pichler, M. S. A. Thompson, and G. Woodward. 2019. A simple model predicts how warming simplifies wild food webs. Nature Climate Change, **9**:611–616.

Parker, S. M. and A. D. Huryn. 2006. Food web structure and function in two arctic streams with contrasting disturbance regimes. Freshwater Biology, **51**:1249–1263.

Paviour-Smith, K. 1956. The biotic community of a salt meadow in New Zealand. Transactions of the Royal Society of New Zealand, **83**:525–554.

Percival, E. and H. Whitehead. 1929. A quantitative study of the fauna of some types of stream-bed. Journal of Ecology, **17**:282–314.

Peterson, C. H. 1979. The importance of predation and competition in organizing the intertidal epifaunal communities of Barnegat Inlet, New Jersey. Oecologia, **39**:1–24.

Piechnik, D. A., S. P. Lawler, and N. D. Martinez. 2008. Food-web assembly during a classic biogeographic study: species'"trophic breadth" corresponds to colonization order. Oikos, **117**:665–674.

Poepperl, R. 2003. A quantitative food web model for the macroinvertebrate community of a northern German lowland stream. International Review of Hydrobiology, **88**:433–452.

Preston, D. L., S. A. Orlofske, J. P. McLaughlin, and P. T. J. Johnson. 2012. Food web including infectious agents for a California freshwater pond. Ecology, **93**:1760–1760.

Ratsirarson, J. and J. A. Silander. 1996. Structure and dynamics in Nepenthes madagascariensis pitcher plant micro-communities. Biotropica, **28**:218–227.

Richards, O. W. 1926. Studies on the ecology of English Heaths: III. Animal communities of the felling and burn successions at Oxshott Heath, Surrey. Journal of Ecology, **14**:244–281.

Ricker, W. E. 1934. An ecological classification of certain Ontario streams. In Publications of the Ontario Fisheries Research Laboratory, No. 49, pages 7–114. Biological Series, No. 37, University of Toronto, CAN.

Rosenthal, R. J., W. D. Clarke, and P. K. Dayton. 1974. Ecology and natural history of a stand of giant kelp, Macrocystis pyrifera, off Del Mar, California. Fishery Bulletin, **72**:670–684.

Schneider, D. W. 1997. Predation and food web structure along a habitat duration gradient. Oecologia, **110**:567–575.

Schröter, D., V. Wolters, and P. C. De Ruiter. 2003. C and N mineralisation in the decomposer food webs of a European forest transect. Oikos, **102**:294–308.

Smirnov, N. N. 1961. Food cycles in sphagnous bogs. Hydrobiologia, **17**:175–182.

Smith, W. O., D. G. Ainley, and R. Cattaneo-Vietti. 2007. Trophic interactions within the Ross Sea continental shelf ecosystem. Philosophical Transactions of the Royal Society B: Biological Sciences, **362**:95–111.

Stagliano, D. M. and M. R. Whiles. 2002. Macroinvertebrate production and trophic structure in a tallgrass prairie headwater stream. Journal of the North American Benthological Society, **21**:97–113.

Stewart, T. J. and W. G. Sprules. 2011. Carbon-based balanced trophic structure and flows in the offshore lake Ontario food web before (1987–1991) and after (2001–2005) invasion-induced ecosystem change. Ecological Modelling, **222**:692–708.

Summerhayes, V. S. and C. S. Elton. 1923. Contributions to the ecology of Spitsbergen and Bear Island. Journal of Ecology, **11**:214—286.

Tavares-Cromar, A. F. and D. D. Williams. 1996. The importance of temporal resolution in food web analysis: Evidence from a detritus-based stream. Ecological Monographs, **66**:91–113.

Thompson, R. M., K. N. Mouritsen, and R. Poulin. 2005. Importance of parasites and their life cycle characteristics in determining the structure of a large marine food web. Journal of Animal Ecology, **74**:77–85.

Thompson, R. M. and C. R. Townsend. 2003. Impacts on stream food webs of native and exotic forest: An intercontinental comparison. Ecology, **84**:145–161.

Thompson, R. M. and C. R. Townsend. 2004. Land-use influences on New Zealand stream communities: Effects on species composition, functional organisation, and food-web structure. New Zealand Journal of Marine and Freshwater Research, **38**:595–608.

Tilly, L. J. 1968. The structure and dynamics of Cone Spring. Ecological Monographs, **38**:169–197.

Torres, M. Á., M. Coll, J. J. Heymans, V. Christensen, and I. Sobrino. 2013. Food-web structure of and fishing impacts on the Gulf of Cadiz ecosystem (South-western Spain). Ecological Modelling, **265**:26–44.

Twomey, A. C. 1945. The bird population of an elm-maple forest with special reference to aspection, territorialism, and coactions. Ecological Monographs, **15**:173–205.

Valiela, I. 1969. An experimental study of the mortality factors of larval Musca autumnalis DeGeer. Ecological Monographs, **39**:199–225.

Valiela, I. 1974. Composition, food webs and population limitation in dung arthropod communities during invasion and succession. The American Midland Naturalist, **92**:370–385.

van Es, F. B. 1977. A preliminary carbon budget for a part of the Ems estuary: the Dollard. Helgoländer wissenschaftliche Meeresuntersuchungen, **30**:283–294.

Vinogradov, M. E. and E. A. Shushkina. 1978. Some development patterns of plankton communities in the upwelling areas of the Pacific Ocean. Marine Biology, **48**:357–366.

Warren, P. H. 1989. Spatial and temporal variation in the structure of a freshwater food web. Oikos, **55**:299–311.

Warren, P. H. 1990. Variation in food-web structure: The determinants of connectance. The American Naturalist, **136**:689–700.

Whittaker, P. L. 1984. The insect fauna of mistletoe (Phoradendron tomentosum, Loranthaceae) in southern Texas. The Southwestern Naturalist, **29**:435–444.

Wilbur, H. M. 1972. Competition, predation, and the structure of the Ambystoma-Rana sylvatica community. Ecology, **53**:3–21.

Woodwell, G. M. 1967. Toxic substances and ecological cycles. Scientific American, **216**:24–31.

Yodzis, P. 1998. Local trophodynamics and the interaction of marine mammals and fisheries in the Benguela ecosystem. Journal of Animal Ecology, **67**:635–658.

Zaret, T. M. and R. T. Paine. 1973. Species introduction in a tropical lake. Science, **182**:449–455.

Zetina-Rejón, M. J., F. Arreguín-Sánchez, and E. A. Chávez. 2003. Trophic structure and flows of energy in the Huizache–Caimanero lagoon complex on the Pacific coast of Mexico. Estuarine, Coastal and Shelf Science, **57**:803–815.
